# Diversification of gene content in the *Mycobacterium tuberculosis* complex is determined by phylogenetic and ecological signatures

**DOI:** 10.1128/spectrum.02289-23

**Published:** 2024-01-17

**Authors:** Taiana Tainá Silva-Pereira, Naila Cristina Soler-Camargo, Ana Marcia Sá Guimarães

**Affiliations:** 1 Laboratory of Applied Research in Mycobacteria, Department of Microbiology, Institute of Biomedical Sciences, University of São Paulo, São Paulo, Brazil; 2 Department of Preventive Veterinary Medicine and Animal Health, School of Veterinary Medicine and Animal Sciences, University of São Paulo, São Paulo, Brazil; University of Arkansas for Medical Sciences, Little Rock, Arkansas, USA

**Keywords:** *Mycobacterium tuberculosis*, genomics, pan-genome, *Mycobacterium bovis*, *Mycobacterium africanum*, evolution

## Abstract

We analyzed the pan-genome and gene content modulation of the most diverse genome data set of the *Mycobacterium tuberculosis* complex (MTBC) gathered to date. The closed pan-genome of the MTBC was characterized by reduced accessory and strain-specific genomes, compatible with its clonal nature. However, significantly fewer gene families were shared between MTBC genomes as their phylogenetic distance increased. This effect was only observed in inter-species comparisons, not within-species, which suggests that species-specific ecological characteristics are associated with changes in gene content. Gene loss, resulting from genomic deletions and pseudogenization, was found to drive the variation in gene content. This gene erosion differed among MTBC species and lineages, even within *M. tuberculosis*, where L2 showed more gene loss than L4. We also show that phylogenetic proximity is not always a good proxy for gene content relatedness in the MTBC, as the gene repertoire of *Mycobacterium africanum* L6 deviated from its expected phylogenetic niche conservatism. Gene disruptions of virulence factors, represented by pseudogene annotations, are mostly not conserved, being poor predictors of MTBC ecotypes. Each MTBC ecotype carries its own accessory genome, likely influenced by distinct selective pressures such as host and geography. It is important to investigate how gene loss confer new adaptive traits to MTBC strains; the detected heterogeneous gene loss poses a significant challenge in elucidating genetic factors responsible for the diverse phenotypes observed in the MTBC. By detailing specific gene losses, our study serves as a resource for researchers studying the MTBC phenotypes and their immune evasion strategies.

**IMPORTANCE:**

In this study, we analyzed the gene content of different ecotypes of the *Mycobacterium tuberculosis* complex (MTBC), the pathogens of tuberculosis. We found that changes in their gene content are associated with their ecological features, such as host preference. Gene loss was identified as the primary driver of these changes, which can vary even among different strains of the same ecotype. Our study also revealed that the gene content relatedness of these bacteria does not always mirror their evolutionary relationships. In addition, some genes of virulence can be variably lost among strains of the same MTBC ecotype, likely helping them to evade the immune system. Overall, our study highlights the importance of understanding how gene loss can lead to new adaptations in these bacteria and how different selective pressures may influence their genetic makeup.

## INTRODUCTION

The *Mycobacterium tuberculosis* complex (MTBC) causes tuberculosis (TB) in humans and animals, a transmissible disease that leads to 1.6 million human deaths every year (1) and inestimable losses in livestock and wildlife (2, 3). The MTBC is composed of 11 species or ecotypes with variable host tropism and virulence, classified into human- or animal-adapted strains (4–6). Accordingly, *Mycobacterium tuberculosis sensu stricto* (lineages L1–L4, L7, and L8) and *Mycobacterium africanum* (lineages L5, L6, and L9) are largely responsible for the TB burden in humans and highly adapted to this host species (6–10). *Mycobacterium bovis* and, to a lesser extent, *Mycobacterium caprae* are the main animal-adapted strains responsible for zoonotic, livestock, and wildlife TB (11–13). Compared to *M. tuberculosis*, *Mycobacterium bovis* has a broader host tropism (6), being able to infect and establish animal reservoirs in different host species with variable populational persistence (6, 14). More recently, the animal-adapted strain *Mycobacterium orygis* has been suggested as a pathogen of zoonotic TB in South Asia (15). Other animal-adapted MTBC species include: *Mycobacterium mungi*, *Mycobacterium pinnipedii*, *Mycobacterium microti*, “dassie bacillus,” *Mycobacterium suricattae*, and “chimpanzee bacillus.” These species have been mostly associated with the primary host from which they were first isolated, but *M. pinnipedii* and *M. microti* were described infecting humans in spill-over events (16–22). Except for *M. bovis*, the true host range and virulence of animal-adapted MTBC species is poorly described.

Members of the MTBC are under clonal evolution and have very similar genomes, represented by >99.9% identity over homologous regions. Horizontal gene transfers (HGT), large recombinations, and significant variations in gene synteny are absent (4, 23, 24). Genetic differences among MTBC genomes are given by single nucleotide polymorphisms (SNPs), small indels (insertions and deletions), large deletions of up to ~15 Kb, and insertion of transposable elements (insertion sequences, IS) (4, 24, 25). Despite this high similarity, the different species and lineages of the MTBC acquired adaptive traits throughout evolution to differentially survive in various hosts, environment, and to modulate virulence.

Because of their clonality and absence of HGT, genetic variability in the MTBC is expected to be limited. However, prior analyses of gene content in two clonal pathogens, *M. tuberculosis* and *Yersinia pestis*, have revealed that gene gain and/or loss play a significant role in generating genetic diversity in these bacterial species, surpassing initial estimates (26). Accordingly, MTBC genes are lost over time due to genomic decay caused by genomic deletions and gene disruptions (26–29). Our research group has recently demonstrated that gene disruptions in *M. tuberculosis*, *M. africanum*, and *M. bovis* predominantly result from indels causing frameshift mutations, SNPs causing non-sense mutations, or the insertion of mobile elements (e.g., IS) (29). This remodeling of gene content associated with mutations altering gene function in the MTBC may be the reason why individual species and lineages display distinct host range and virulence phenotypes, thereby warranting further studies.

Gene gain and loss can only be estimated through the evaluation of gene content of individual strains at the population level (30). Hence, pan-genome analysis constitutes an important tool to estimate the diversity of the gene repertoire of defined bacterial groups (31). The vast majority of MTBC comparative genomic studies are focused on the evaluation of SNPs and indels (32–47). Studies analyzing gene content of the MTBC considered strains of *M. tuberculosis*, *M. africanum*, *M. bovis*, and/or *M. microti* (28, 38, 48–54). Other MTBC species/ecotypes were not comparatively analyzed within the complex. There are also contradictory reports on the openness of the *M. tuberculosis* or MTBC pan-genome (52, 55–57). Therefore, the aims of this study were to assess the pan-genome of MTBC using available representatives from all described species of the complex and evaluate the impact of gene loss and gain on virulence factors (VFs) and metabolism of the MTBC. This study constitutes the most diverse pan-genome analysis of the MTBC described to date in terms of human- and animal-adapted strains.

## MATERIALS AND METHODS

### Data set of MTBC genomes

All complete genomes of the MTBC (except for Bacillus Calmette-Guérin strains) available in RefSeq (Reference Sequence database), NCBI (National Center for Biotechnology Information), as of September 2022 were retrieved, totalizing 310 genomes. Among these, the vast majority (*n* = 296) were of *M. tuberculosis*. Thus, to increase the number of genomes from other MTBC species, both complete and draft genomes *of M. africanum, M. bovis, M. caprae, M. mungi, M. pinnipedii, M. orygis, M. microti,* and “dassie bacillus” available in RefSeq were included, while maintaining only complete genomes of *M. tuberculosis*. For all genomes, the latest PGAP (Prokaryotic Genome Annotation Platform) annotation available was retrieved for this study.

To be deposited in RefSeq, a genome must already meet several quality criteria determined by the NCBI. Nevertheless, for genomes to be included in our analyses, additional criteria were established: (i) being sequenced in Illumina platforms or a combination of Illumina and gap closure with Sanger (i.e., genomes sequenced with 454, Ion Torrent, PacBio, or only-Sanger were excluded); (ii) being annotated by PGAP; (iii) having less than 300 contigs and N50 >20,000 bp (56); and (iv) having >95% completeness against species-specific reference strains according to BUSCO v5.4.3 (58). Therefore, 114 genomes of *M. tuberculosis* (all complete/closed), 33 genomes of *M. africanum* (1 complete and 32 drafts), 66 genomes of *M. bovis* (1 complete and 65 drafts), 4 draft genomes of *M. caprae*, 1 draft genome of *M. mungi*, 3 draft genomes of *M. pinnipedii*, 2 draft genomes of *M. orygis*, 6 genomes (5 complete and 1 draft) of *M. microti*, and 4 draft genomes of “dassie bacillus” were retrieved, totalizing 233 MTBC genomes (Fig. S1; Table S1). Unfortunately, genomes of *M. suricattae* and “chimpanzee bacillus” were available only as reads, i.e., annotated assemblies were not publicly accessible at the time of this study (53, 59). Table 1 provides statistics on the number of contigs and median N50 of draft genomes.

**TABLE 1:** Number of contigs of the selected draft genomes of the *Mycobacterium tuberculosis* complex (*n* = 112)

	Number of contigs			Contig N50 values		
Species	Minimum	Median	Maximum	Minimum	Median	Maximum
*M. africanum* (*n* = 32)	5	19	194	68,069	464,945	1,456,982
*M. bovis* (*n* = 65)	8	98	239	29,930	105,873	3,667,979
*M. caprae* (*n* = 4)	2	124	177	80,893	94,807	3,897,638
*M. mungi* (*n* = 1)	–^*a*^	110	–	–	97,561	–
*M. pinnipedii* (*n* = 3)	116	118	226	51,414	65,266	68,061
*M. orygis* (*n* = 2)	107	108	108	97,089	98,423	99,756
*M. microti* (*n* = 1)	–	259	–	–	27,952	–
“dassie bacillus” (*n* = 4)	193	227	260	43,105	46,105	57,397

^*a*^ –, no minimum or maximum number of contigs because there is only one draft genome each of *M. mungi* and *M. microti*.

### Lineage identification and core SNP phylogeny

A phylogenetic tree of the MTBC genomes was generated using kSNP3 (60) to aid in lineage identification and species confirmation. This software was chosen because it accepts assembled genomes as input, since reads were not available for all analyzed MTBC strains. Briefly, core polymorphic sites were identified using default parameters in kSNP3 and the core SNP matrix was then subjected to ascertainment bias correction (ASC) using the “-fconst” directive of IQ-Tree (61) as described (39). The model selection program implemented in IQ-Tree was used to select the best substitution model for the ASC-corrected SNP alignment according to Bayesian Information Criterion. The best model, HKY + I + G (which was used to model transition/transversion rates and unequal base frequency), was then fixed for the maximum likelihood (ML) phylogenetic reconstruction using 1,000 UFBoot pseudoreplicates (62). Visualization and customization of the phylogenetic tree were performed using FigTree (63) and Adobe Illustrator software. *Mycobacterium canettii* CIPT140010059 was used as an outgroup. Lineage information available from metadata of each BioSample and from a previous study that included selected genomes (29), along with a lineage identification methodology previously described (29), were used to confirm the lineages.

### Pan-genome analysis with and without predicted pseudogenes

Proteome files of each genome were retrieved from RefSeq [i.e., protein.faa, which contains proteins from predicted CDS (coding DNA sequence), and translated_cds.faa, which contains proteins from predicted CDS and PGAP-detected pseudogenes]. The translated_cds.faa file was modified to be used in this study. Briefly, the PGAP pipeline annotates incomplete genes at the end of contigs from draft genomes as pseudogenes. Thus, a previous script (Pseudo_retriev.py) (29) was used to remove these false pseudogenes from the data set. Next, the proteomes (with or without proteins from pseudogenes, separately) were clustered into homologous groups using OrthoFinder (64) with default parameters. The identified groups were used to construct pan-genomes of the analyzed MTBC strains, separated into core [i.e., proteins present in at least 95% (≥221 strains) of the analyzed strains], accessory genome (i.e., proteins present in 2 up to 221 MTBC strains), and strain-specific genome (proteins or group of orthologous proteins present in only one genome).

PCA (principal component analysis) of shared orthologous proteins among the MTBC species was performed using prcomp in R software version 4.1.3 (65). In addition, orthologous protein groups of the core and accessory genomes were used as inputs to a customized script (accessory_matrix.py) (29) that investigate the presence (“1”) and absence (“0”) of each cluster in each genome to generate matrix of 0’s and 1’s. This matrix was then converted into a heatmap using ggplot2 (66) in R software version 4.1.3 (65).

### Estimation of pan-genome openness or closeness

The refined power-law pan-genome model (67) was applied to define the openness or closeness of the MTBC pan-genome. Briefly, the number (*n*) of new genes is plotted for increasing values of the number (*N*) of genomes using the orthologous protein groups identified with OrthoFinder as input in micropan (68) in R software version 4.1.3 (65). If the exponent alpha is >1, that bacterial group has a closed pan-genome.

### SNP distance and Gene Repertoire Relatedness (GRR) index

The core SNP matrix generated by kSNP3 was used as input to calculate the SNP distance between each pair of genomes using ape (69) and plotted with ggplot2 (66) in R software version 4.1.3 (65). The GRR index between each pair of genomes was calculated using a pairwise matrix of shared gene families obtained from the OrthoFinder analysis as described (70). GRR is defined as the number of common gene families between two genomes divided by the number of CDS of the smallest genome (70). Patristic distances between genomes (i.e., the sum of branch lengths in the path between two genomes in the ML phylogenetic tree) was also calculated using ape (69). Results from GRR and patristic distance were plotted with ggplot2 (66) and analyzed with linear regression and generalized additive model smoothing method using the mgcv v.1.8.23 (https://cran.r-project.org/web/packages/mgcv/index.html) in R software version 4.1.3 (65).

### Pan-genome and functional potential of the MTBC species

Functional class annotation of groups of orthologous proteins was performed using EggNOG-mapper version 2 (71), which classifies each protein based on the Cluster of Orthologous Groups (COG) (72). Enrichment analysis of the COGs was performed by comparing the proportion of each functional class observed in the core, accessory, and strain-specific genomes against the observed proportions of the pan-genome as a whole. Pearson’s χ^2^ test was used to identify COGs with statistically significant divergence (*P*-value ≤0.05), with correction for multiple comparisons.

To perform inter-species comparisons, the core genome, accessory genome, and strain-specific genome were identified as described above in separate groups containing all strains of *M. tuberculosis* (G1; *n* = 114), *M. africanum* (G2; *n* = 33), *M. bovis* (G3; *n* = 66), and a group of “animal strains” (G4; *n* = 20) consisting of genomes of *M. caprae, M. mungi, M. pinnipedii*, *M. orygis*, *M. microti*, and “dassie bacillus.” The non-*M*. *bovis* animal strains were grouped due to the low number of genomes. These pan-genomes were then classified into COG and an enrichment analysis was performed as described. In addition, groups of orthologous proteins of the accessory genomes of each bacterial group (excluding hypothetical proteins) were analyzed with STRING v. 11.5 (73) to obtain protein networks.

### Virulence factors

A multifasta file of the protein sequences of 227 VFs of *Mycobacterium* spp. was retrieved from the VFDB (virulence factor database) (74). According to VFDB, VFs refer to the properties (i.e., gene products) that enable a microorganism to establish itself on or within a host of a particular species and enhance its potential to cause disease. This file was used as an input into the Orthofinder tool (64) along with the proteome of each strain. The presence of a given VF in a genome was detected when its protein sequence clustered with its corresponding VFDB sequence. To identify if the absence of a gene was due to pseudogenization (i.e., the gene was annotated as a pseudogene by PGAP parameters), a pseudogene DNA file of each genome was created as described previously (29). The multifasta file of VFs was then searched against this pseudogene DNA data set using tblastn with ≥95% identity and ≥80% query coverage parameters (29). The presence and absence of VFs were displayed in a heatmap generated using ggplot2 in R software version 4.1.3 (65). Paralogous VFs were not considered. Regions of difference (RDs) were retrieved from a previous study (75).

## RESULTS AND DISCUSSION

### Phylogenetic reconstruction and characterization of the data set

**Fig 1:**
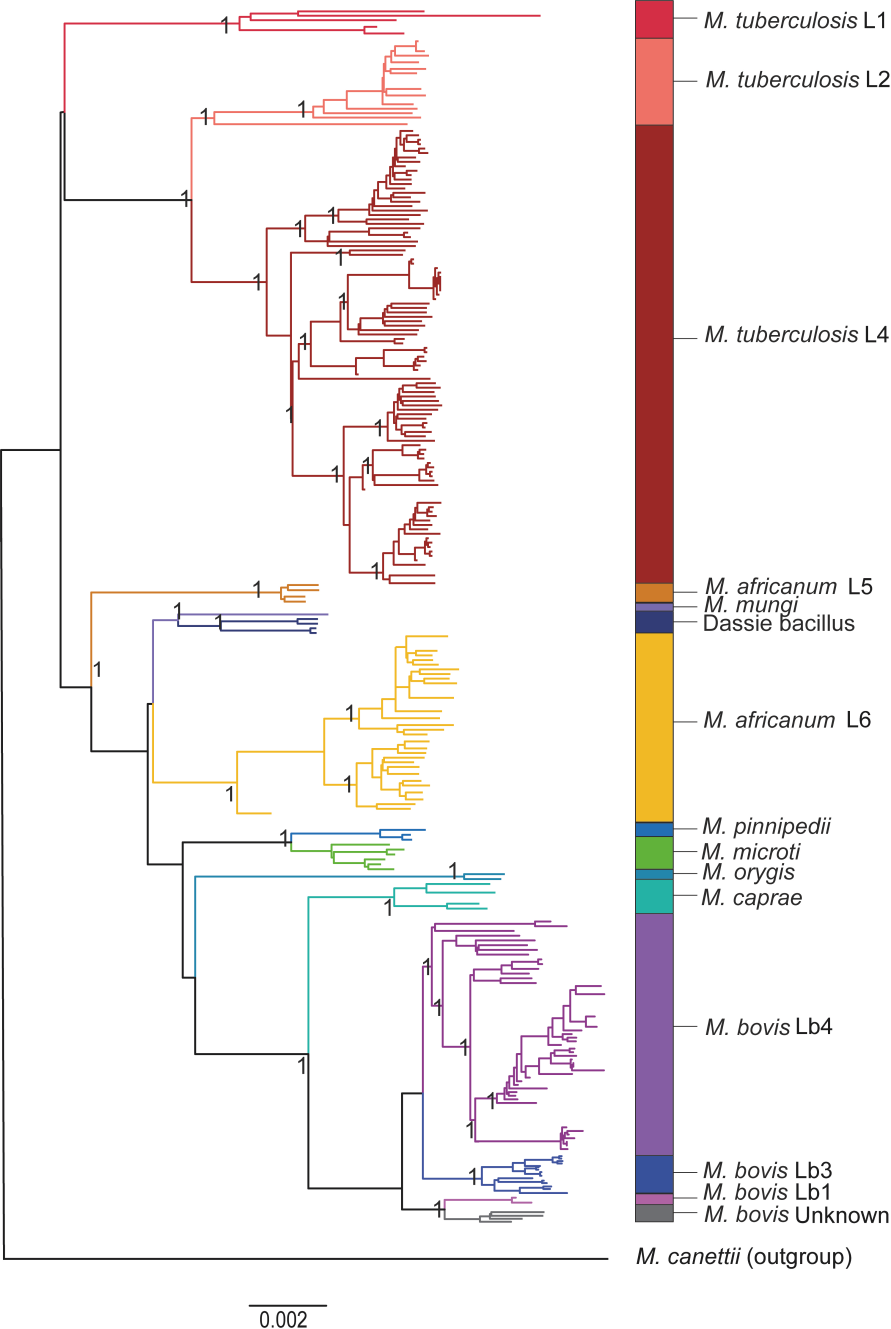
Maximum likelihood phylogenetic tree based on core SNPs of the 233 genomes of the MTBC used in this study. Colored branches correspond to bacterial species and lineages. *Mycobacterium canettii* CIPT 140010059 was used as an outgroup. Genome representatives of *M. tuberculosis* L3, L7, and L8 and *M. africanum* L9 were not detected in the data set, hence not included in the study. A core SNP matrix was generated using kSNP3 (60) and the phylogenetic tree was inferred using IQ-Tree with 1,000 UFBoot pseudoreplicates. Graphical edition was performed using FigTree (63) and Adobe Illustrator software. Bootstrap replicas of main nodes are all ≥90%. Bar shows substitutions per nucleotide.

A total of 233 MTBC genomes deposited in RefSeq, NCBI, and annotated with PGAP were selected following specific quality criteria of sequencing methodology, completeness, and maximum number of contigs. The phylogenetic relationships among these 233 MTBC genomes are shown in Fig. 1. Of the 114 *M*. *tuberculosis* genomes, 5 (4.39%) were identified as *M. tuberculosis* L1, 12 (10.53%) as *M. tuberculosis* L2, and 97 (85.09%) as *M. tuberculosis* L4. Four (12.12%) out of the 33 *M*. *africanum* genomes were identified as L5 and 29 (87.88%) as L6. Genome representatives of *M. tuberculosis* L3, L7, and L8 and *M. africanum* L9 were not found in the data set. A recent genome-based classification system of *M. bovis* was used (39). Of the 66 *M*. *bovis* genomes, 2 (3.03%) were identified as *M. bovis* Lb1, 15 (22.73%) as *M. bovis* Lb3, 45 (68.18%) as *M. bovis* Lb4, and 4 (6.06%) as unknown.

### Pan-genome of the MTBC

The 961,858 proteins of the 233 MTBC genomes generated a pan-genome composed of 3,478 groups of orthologous proteins (*n* ≥ 2 proteins/group, total: 961,273 proteins) and 585 strain-specific proteins (i.e., present in only one genome). The number of groups of orthologous proteins and strain-specific proteins is only about 1.01 times the average number of CDS in an MTBC genome (~4,000 genes per genome, ranging from 3,666 to 4,626 genes), suggesting overall low genetic diversity among strains compared to bacterial species that undergo HGT (70).

**Fig 2:**
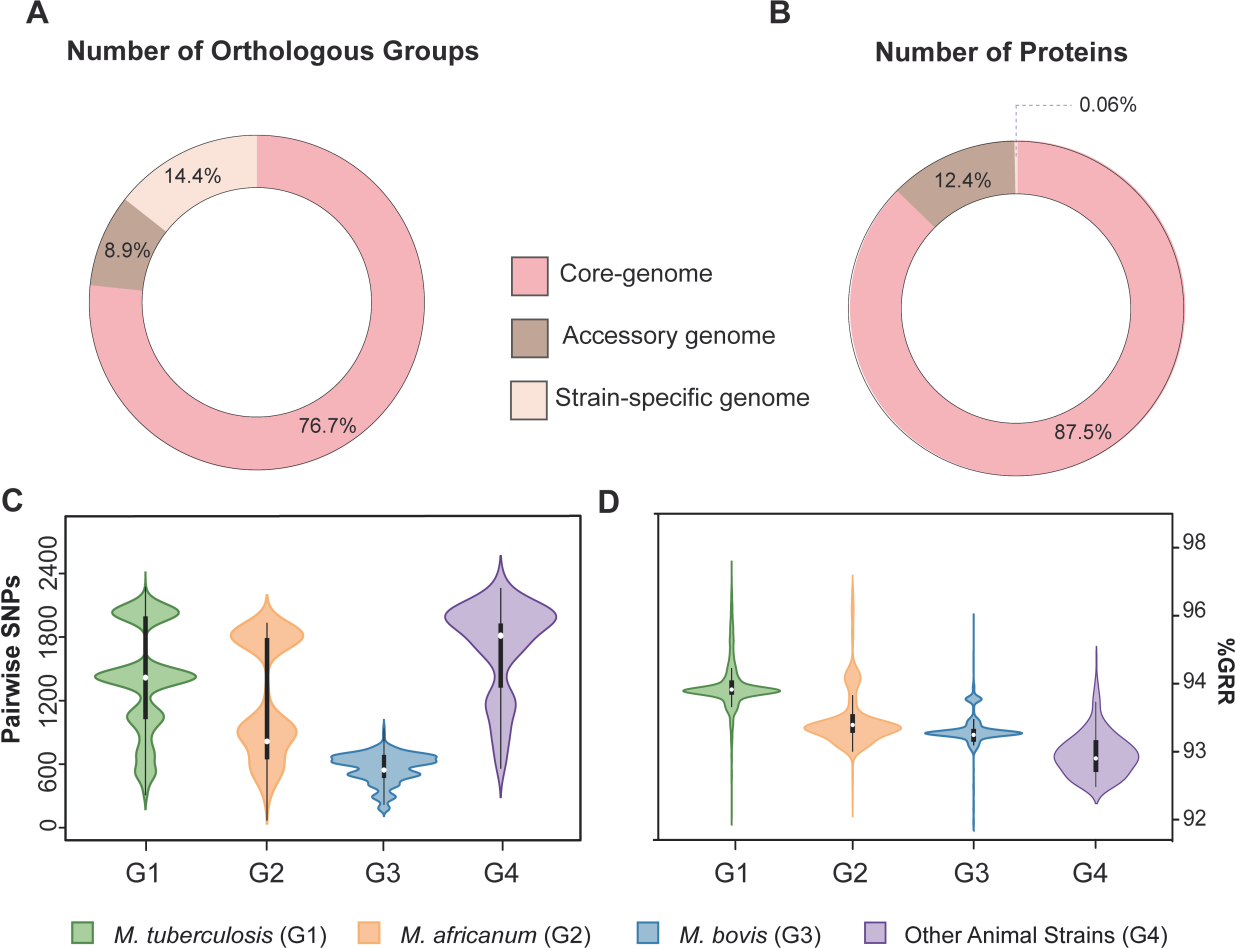
Pan-genome distributions, SNP distance, and %GRR between genomes of 233 strains of the MTBC. (**A**) Percentage distribution of the groups of orthologous proteins in core, accessory, and strain-specific genomes. (**B**) Percentage distribution of the total number of proteins in core, accessory, and strain-specific genome. Pseudogenes are not included in this analysis. (**C**) Violin plot of the SNP distance between genomes of each bacterial group. (**D**) Violin plot of the %GRR between genomes of each bacterial group. Groups are *Mycobacterium tuberculosis* (G1, *n* = 114), *Mycobacterium africanum* (G2, *n* = 33), *Mycobacterium bovis* (G3, *n* = 66), and animal strains (G4, *n =* 20). Animal strains include *Mycobacterium caprae*, *Mycobacterium mungi*, *Mycobacterium pinnipedii*, *Mycobacterium orygis*, *Mycobacterium microti*, and “dassie bacillus.” Genome representatives of *M. tuberculosis* L3, L7, and L8 and *M. africanum* L9 were not detected in the data set, hence not included in the study. No statistical difference was observed among groups (*P*-value >0.05) using Mann-Whitney test. Graphs were generated using ggplot2 in R software version 4.1.3 (65).

Out of the 4,063 groups of proteins of the pan-genome, 3,116 groups (76.69%; total proteins: 841,716, 87.51%) are part of the core genome, while 362 groups of orthologous proteins (8.91%; total proteins: 119,557, 12.43%) are part of the accessory genome, and 585 are strain-specific proteins (14.40%; 0.06%) (Fig. 2). These results reflect the clonal nature and high genomic identity of the MTBC, with most proteins (87.51%) present in >95% of the analyzed genomes.

### The impact of disrupted gene annotations on pan-genome predictions

Throughout evolution, genes can be disrupted by frameshifts, large deletions, insertion of transposable elements, among others (29). Gene prediction and annotation platforms may or may not report these disrupted sequences as pseudogenes, which may influence pan-genome calculations. PGAP has a specialized pseudogene detection tool and reports pseudogenes with a “pseudo” qualifier. While their predicted protein sequences are not included in the final proteomes of genomes, the sequences are listed in the translated_cds.faa file (29). Thus, we analyzed the impact of *in silico* predicted pseudogenes on pan-genome estimates of the MTBC using the protein sequences from this file. The median number of pseudogenes/genome as reported by PGAP in this data set was 140. When analyzing proteomes predicted from pseudogenes and genes, the core genome decreased from 77% to 71% compared to the proteome of genes only (Fig. S2). This percentual reduction occurred mainly due to an increase in strain-specific proteins from 14% to 20% (Fig. S2). These results indicate that MTBC proteins predicted from pseudogenes are mostly not homologous. Using annotation platforms that do not distinguish pseudogenes from genes can overestimate the MTBC’s pan-genome, possibly explaining contradictory results in the literature (38, 48–54). As we curated our data set to remove false pseudogenes at the end of contigs (i.e., broken genes; see Materials and Methods), this overestimation could be even higher without this correction.

### The MTBC has a closed pan-genome

By using a power-law pan-genome model (67), the analyzed MTBC data set showed a closed pan-genome (alpha parameter: 1.5), indicative of its clonal nature (Fig. S3). Even when including pseudogenes, the pan-genome remained closed (alpha parameter of 1.5). Therefore, the whole gene diversity of MTBC is entailed in the data set analyzed herein. This result is in agreement with recent pan-genome analyses of *M. tuberculosis* strains (55, 57), but in contrast to others showing an open MTBC’s pan-genome when including other species (50, 52, 56). These opposing studies differed from our study in sample size, the inclusion of *M. canettii* genomes, and/or choice of gene predictor and annotator (e.g., RAST and prodigal + EggNOG/BLAST2GO). Thus, contrasting findings may be explained by differences in strain selection, as *M. canettii* is not part of the MTBC, and/or the use of annotation platforms that do not curate for pseudogenes or broken genes at the end of contigs.

### Genetic distances and GRR of MTBC species

To provide a basic measure of genetic diversity, we computed the pairwise SNP distances within each of the four groups: *M. tuberculosis* (G1), *M. africanum* (G2), *M. bovis* (G3), and all genomes of the remaining animal-adapted MTBC strains (*M. caprae*, *M. mungi*, *M. pinnipedii*, *M. orygis*, *M. microti*, and “dassie bacillus”) (G4) (Fig. 2C). Although there was no significant difference among groups (*P*-value >0.05), a lower median SNP distance is observed in *M. bovis* (G3) when compared to *M. tuberculosis* (G1) and *M. africanum* (G2) (Fig. 2C). This is consistent with the fact that *M. bovis* is a more recently diverged species (39). The “other animal strains” (G4) showed the highest median SNP distance among all bacterial groups, which can be explained by their phylogenetic divergence and broad distribution along the phylogenetic tree of the MTBC.

The violin plots of pairwise SNP distances showed multimodal distributions for all bacterial groups (Fig. 2C). For *M. tuberculosis* and *M. africanum*, this can be explained by higher SNP distances obtained with inter-lineages comparisons (*M. tuberculosis*: L2 versus L4, L1 versus L4, L1 versus L2; *M. africanum*: L5 versus L6) and lower SNP distances obtained with intra-lineages comparisons (*M. tuberculosis*: L1, L2, L4; *M. africanum*: L5, L6) (Fig. S4A and S4B). The bimodal distribution of the pairwise SNP distance of the “other animal strains” (Fig. 2C) also corresponds to inter-species (higher SNP distances) and intra-species (lower SNP distances) comparisons. While a multimodal distribution is also observed for *M. bovis*, the ranges of the pairwise SNP distances are smaller, and mode separation is not as marked as in other bacterial groups (Fig. 2C). Indeed, inter-lineage bimodal distributions of pairwise SNP distance were not markedly evident for *M. bovis* strains (Fig. S4C). Strains of *M. bovis* also showed lower overall pairwise SNP distance (maximum of 848 SNPs) when compared to *M. tuberculosis* and *M. africanum* (maximum of 1,988 and 1,796 SNPs, respectively). Taken together, these results suggest that *M. bovis* strains constitute a more genetically homogeneous population when compared to human-adapted species.

Using the pan-genome matrix, we calculated the GRR between MTBC genomes of each group (i.e., the number of shared-gene families between two genomes) (Fig. 2D). Although no significant difference in the median %GRR among groups was observed (*P*-value >0.05), there was an important amplitude in the %GRR distribution within each group. These marked amplitudes were driven by inter-lineages comparisons (Fig. S5), especially for *M. tuberculosis* and *M. africanum*. In *M. bovis*, the Lb4 intra-lineage comparison also showed a marked amplitude (Fig. S5C).

Genomes of *M. tuberculosis* presented the highest median %GRR, followed by *M. africanum*, *M. bovis,* and “other animal strains” (Fig. 2D). Except for “other animal strains,” a similar trend is seen in the SNP distance distribution among groups (Fig. 2C). Thus, gene repertoire differences cannot be fully explained by SNP divergence between strains, as *M. tuberculosis* strains showed the highest gene repertoire similarity (high %GRR), but the highest SNP distance between genomes. Thus, gene content modulation in MTBC is not driven by SNPs.

### Gene repertoire is associated with currently defined MTBC species

**Fig 3:**
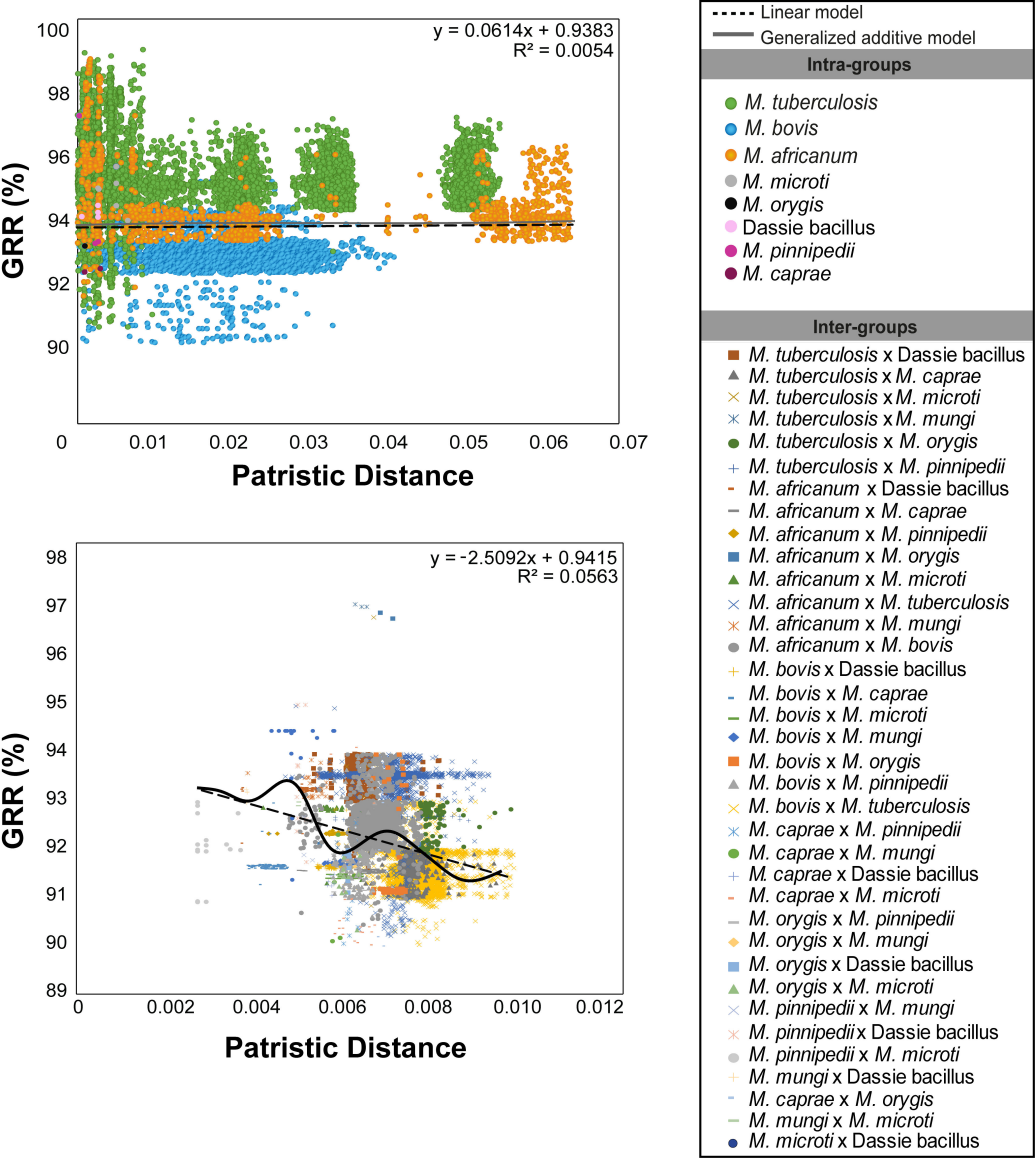
%GRR as a function of the patristic distance between genomes of the MTBC. Top graph shows the correlation between %GRR and patristic distance across pairs of genomes of the same species colored according to each species. MTBC species with only one representative were excluded. Bottom graph shows the correlation between %GRR and patristic distance across pairs of genomes from different species colored according to inter-species comparisons. Graphs were generated using ggplot2 in R software version 4.1.3 (65). Genome representatives of *M. tuberculosis* L3, L7, and L8 and *M. africanum* L9 were not detected in the data set, hence not included in the study.

Current taxonomy of MTBC species is determined by phenotypic characteristics, RD patterns, and SNP-based phylogeny (76). The extent of gene content similarity among current MTBC species or lineages is unknown. Therefore, we evaluated if the shared gene content between genomes of the same species can be predicted by their phylogenetic distance. Surprisingly, intra-species %GRR values do not decrease as a function of the patristic distance between genomes (i.e., sum of the branch lengths of the phylogenetic tree between two genomes) (Fig. 3A). In other words, the phylogenetic distance between strains of the same species is not associated with significant gene content variations. %GRR values only decrease as a function of the patristic distance when performing inter-species comparisons (Fig. 3B), indicating that MTBC’s ecology and lineages play an important role in defining gene repertoire. Thus, phenotypic characteristics historically used to define species of the MTBC, such as host tropism, accompany significant changes in gene content.

**Fig 4:**
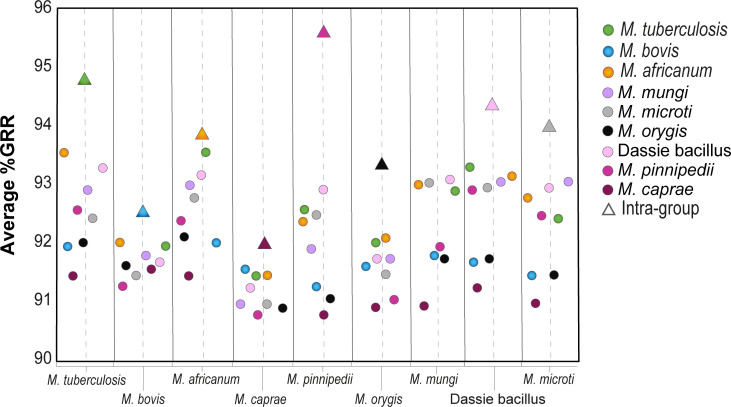
Average %GRR between genomes of the MTBC. Triangles (intra-species) and circles (inter-species) indicate average %GRR in comparison to each species on the x-axis. Genome representatives of *M. tuberculosis* L3, L7, and L8 and *M. africanum* L9 were not detected in the data set, hence not included in the study.

**Fig 5:**
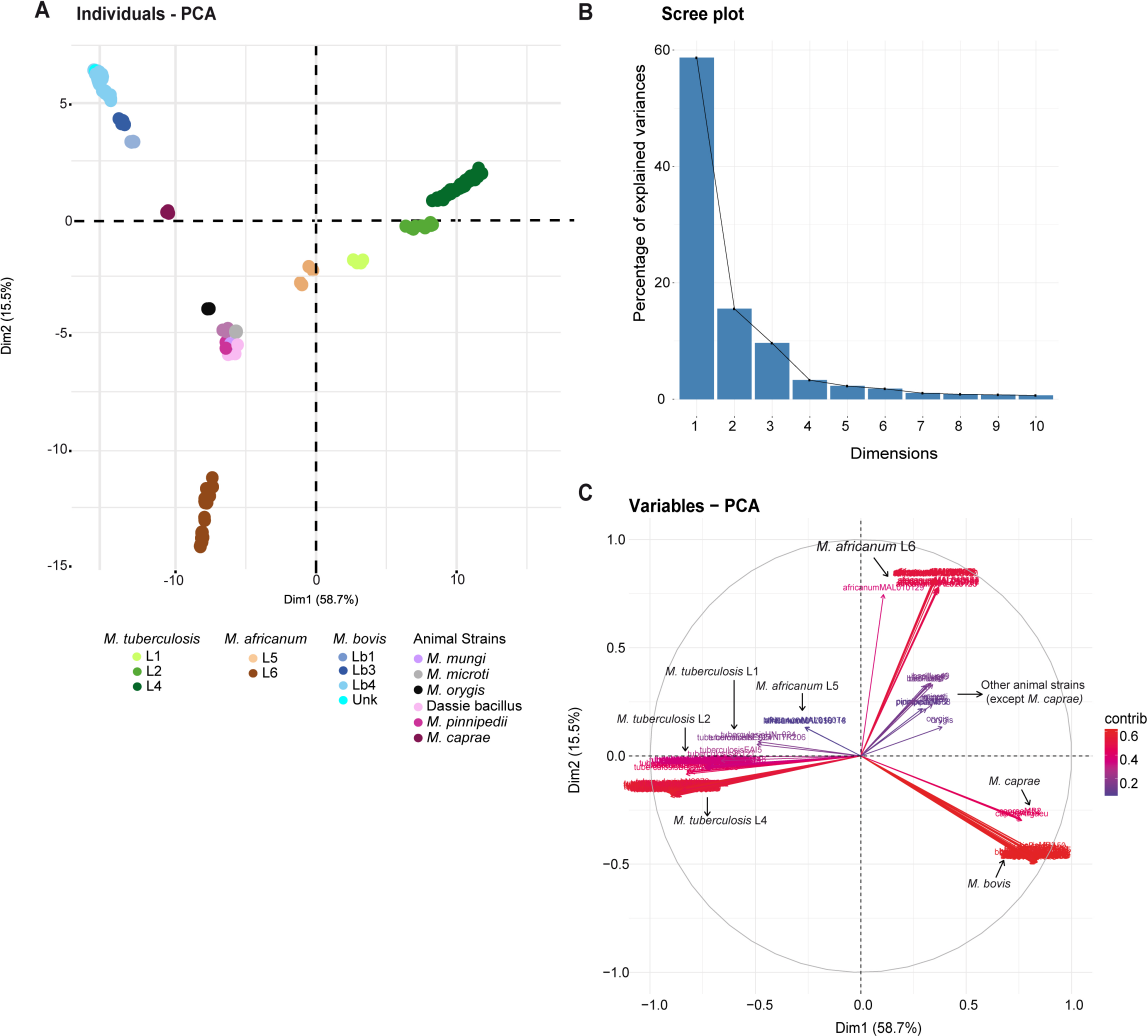
PCA of the groups of orthologous proteins of the MTBC. (**A**) Biplot of the first two principal components, which explain 74.2% of the variance. Lineages and species are colored. (**B**) Proportion (%) of explained variance by each principal component. (**C**) Variables graph of the PCA, zero-centered and scaled to unit variance. PCA was generated using prcomp in R software version 4.1.3. Genome representatives of *M. tuberculosis* L3, L7, and L8 and *M. africanum* L9 were not detected in the data set, hence not included in the study.

Inter-species comparisons of average %GRR showed that *M. africanum* share more gene families with *M. tuberculosis* than with its phylogenetic counterparts *M. mungi* and “dassie bacillus” (Fig. 4; Table S2). Strains of *M. pinnipedii* showed the highest average %GRR (Fig. 4) because two out of three genomes were obtained from the same sea lion and are highly similar (77). PCA of the MTBC’s groups of orthologous proteins indicates that genomes of different species and lineages formed non-overlapping clusters, following overall phylogenetic signatures (Fig. 5A). Accordingly, *M. tuberculosis* L1, L2, and L4 were separated into three groups, while *M. bovis* genomes appeared segregated into groups of Lb1, Lb2, and Lb4 (Fig. 5A), corroborating recent findings about the existence of distinct *M. bovis* lineages (39, 78). A segregation among strains of *M. pinnipedii* was also observed (data not shown) and is in accordance with previously suggested geographical clusters (MP1 and MP2 from South America and ATCC BAA-688 from Australia) (77).

According to our phylogenetic reconstruction (Fig. 1) and previous studies (5, 10), *M. africanum* L6 clusters with *M. mungi* and “dassie bacillus”. Despite this phylogenetic structure, *M. mungi* and “dassie bacillus” appeared closer to *M. microti* and *M. pinnipedii* than to *M. africanum* L6 in the PCA analyses of the matrix of groups of orthologous proteins (Fig. 5). Phylogenetic niche conservatism is defined as the tendency of closely related species to retain similar ecological characteristics throughout their evolutionary history (79). In this context, *M. africanum* L6 deviated from its expected phylogenetic niche conservatism, sharing less gene families with “dassie bacillus” and *M. mungi* than the more distantly related species *M. microti* and *M. pinnipedii*. Throughout evolution, the sustained transmission of *M. africanum* L6 in humans likely shaped its gene repertoire, showing that distinct selective pressures act on gene content compared to the core SNPs used to estimate phylogeny.

The PCA graph also shows that groups of orthologous proteins of *M. tuberculosis* L1 clustered more closely to *M. africanum* L5 than to other *M. tuberculosis* lineages (Fig. 5A). Variables plot of PCs indicates that *M. africanum* L5 is more similar at the gene repertoire level to *M. tuberculosis* lineages than to *M. africanum* L6 or animal-adapted strains (Fig. 5C). Along with L7 (not included), L1, L5, and L6 are considered ancient lineages of the human-adapted strains of the MTBC (80). They are also more restricted geographically than modern lineages L2, L3 (not included), and L4 (80). Thus, even though both *M. africanum* L5 and L6 are ancient lineages, share geography distribution, and carry the same species (or variant) name, they still differ in their gene repertoire.

### Gene loss as a driver of gene content variation

**Fig 6:**
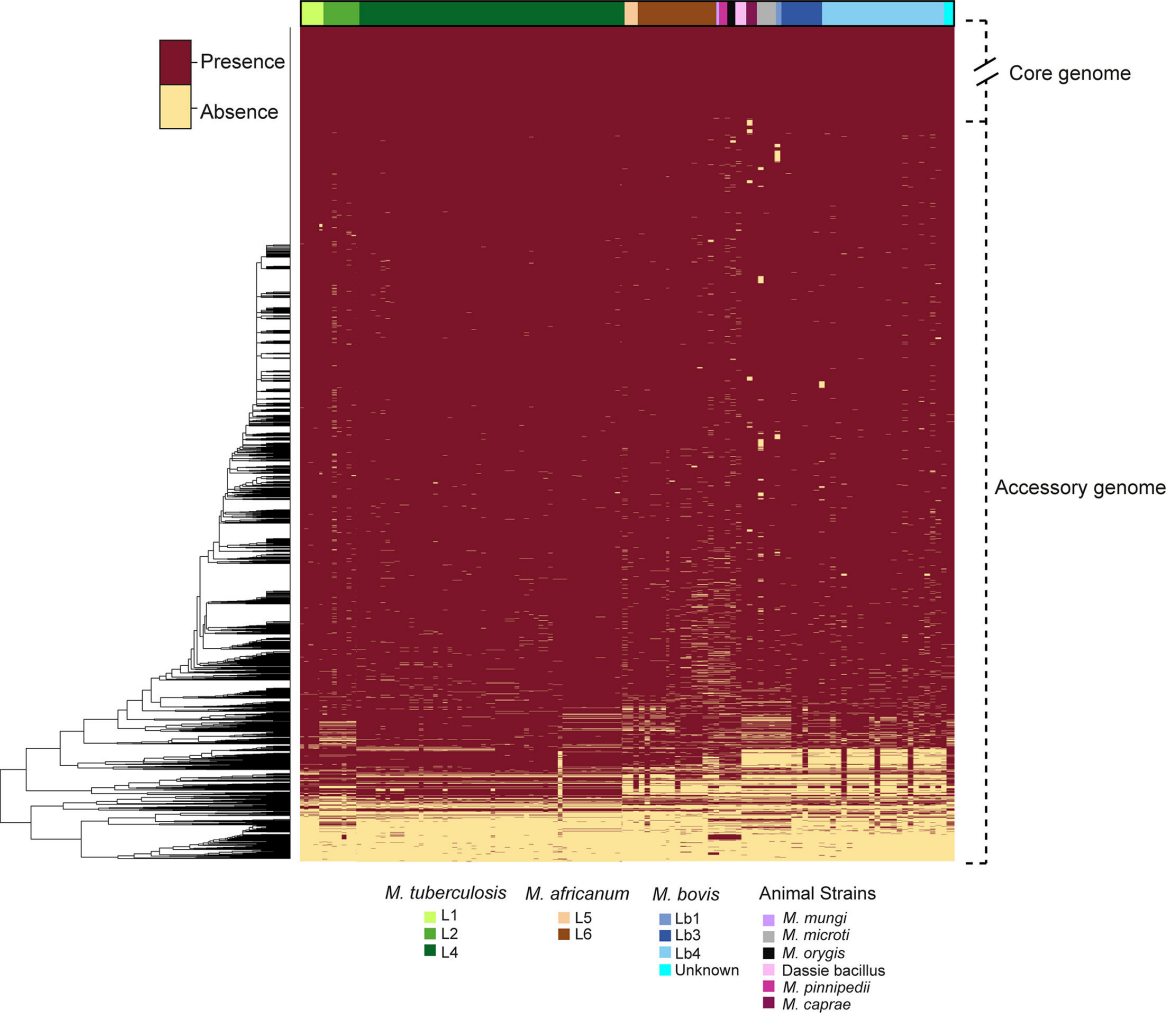
Heatmap of the presence and absence of groups of orthologous proteins of the MTBC. Heatmap was generated with a customized script in Python using the output of OrthoFinder (64) generated from 233 MTBC genomes. Red, protein cluster is present. Yellow, protein cluster is absent. Core genome is shown only partially to simplify the figure. Genome representatives of *M. tuberculosis* L3, L7, and L8 and *M. africanum* L9 were not detected in the data set, hence not included in the study.

To understand the observed variations in %GRR among MTBC species, we constructed a heatmap of the presence and absence of groups of orthologous proteins of the MTBC according to species and lineages (Fig. 6). A significant genomic erosion in genomes of *M. tuberculosis* L2, *M. africanum*, *M. bovis,* and “other animal strains” are observed when compared to *M. tuberculosis* L4. Therefore, gene loss drives the distinct gene repertoire observed among species and lineages of the MTBC.

Strains of *M. tuberculosis* L4 (and possibly *M. tuberculosis* L1) were the least affected by gene erosion (Fig. 6). We have previously described that most pseudogenes are caused by frameshifts due to indels (29). For a mutation to emerge as dominant, there must be prior genotypic heterogeneity in the bacterial population (81, 82). Therefore, it is possible that genotypic heterogeneity caused by indels is higher in non-L4 strains of the MTBC, which could imply more efficient DNA repair mechanisms or lower sensitivity to mutagenic agents or stressors in L4 strains. In contrast, L4 strains may less frequently undergo genetic drift due to population bottlenecks (83) compared to non-L4 strains or be subjected to unique pressures that do not allow specific indels to dominate subpopulations.

### Functional classification of the MTBC pan-genome

To evaluate if there is any functional class associated with gene content variations in the MTBC species, we classified the protein clusters of the pan-genome into COGs. Proteins of unknown function (S) constitute the largest proportion of the MTBC protein clusters, followed by lipid transport and metabolism (I), secondary metabolites biosynthesis, transport, and catabolism (Q), energy production and conversion (C) and amino acid metabolism and transport (E), and others (Fig. S6). While the core genome is over-represented by typical housekeeping functions, the categories of cellular motility (N) and secondary metabolites biosynthesis, transport, and catabolism (Q) stand out as important components of the accessory genome (Fig. S6). Therefore, these classes can be considered hotspots of gene loss throughout MTBC evolution. Proteins classified in the cellular motility category (N) include PE/PPE family, type VII secretion system proteins, and peptidases. Proteins classified in the secondary metabolites biosynthesis, transport, and catabolism (Q) include Mce proteins and polyketide synthases (*pks*).

Eight COG categories of the core genome were the only functional categories significantly enriched compared to the whole pan-genome of the MTBC. These were unknown function (S); lipid transport and metabolism (I); energy production and conversion (C); amino acid transport and metabolism (E); transcription (K); translation, ribosomal structure, and biogenesis (J); RNA processing and modification (A); chromatin structure and dynamics (B) (Fig. S6B). This overall enrichment in housekeeping functions indicates that these categories are less prone to gene loss. None of the functional categories of the accessory genome or strain-specific genome were significantly enriched compared to the whole pan-genome (Fig. S6B).

We also calculated the pan-genome of each bacterial group (G1 to G4, Table 2) and classified their pan-genomes according to COG. Detailed results are shown in Supplementary file 1. Overall, functional classes were differentially affected by gene loss depending on the MTBC species, which suggests distinct selective pressures shaping their gene repertoire.

**TABLE 2:** Pan-genome of the MTBC and bacterial groups^*a*^

Bacterial groups	Protein clusters		Strain-specific
	Core	Accessory	
MTBC	3,116	362	585
*M. tuberculosis* (G1)	3,460	250	281
*M. africanum* (G2)	3,583	154	244
*M. bovis* (G3)	3,341	127	333
Other animal strains (G4)	3,300	155	333

^*a*^ Other animal strains: genomes of *Mycobacterium caprae*, *Mycobacterium mungi, Mycobacterium pinnipedii*, *Mycobacterium orygis, Mycobacterium microti*, and “dassie bacillus.”

### Virulence factors

**Fig 7:**
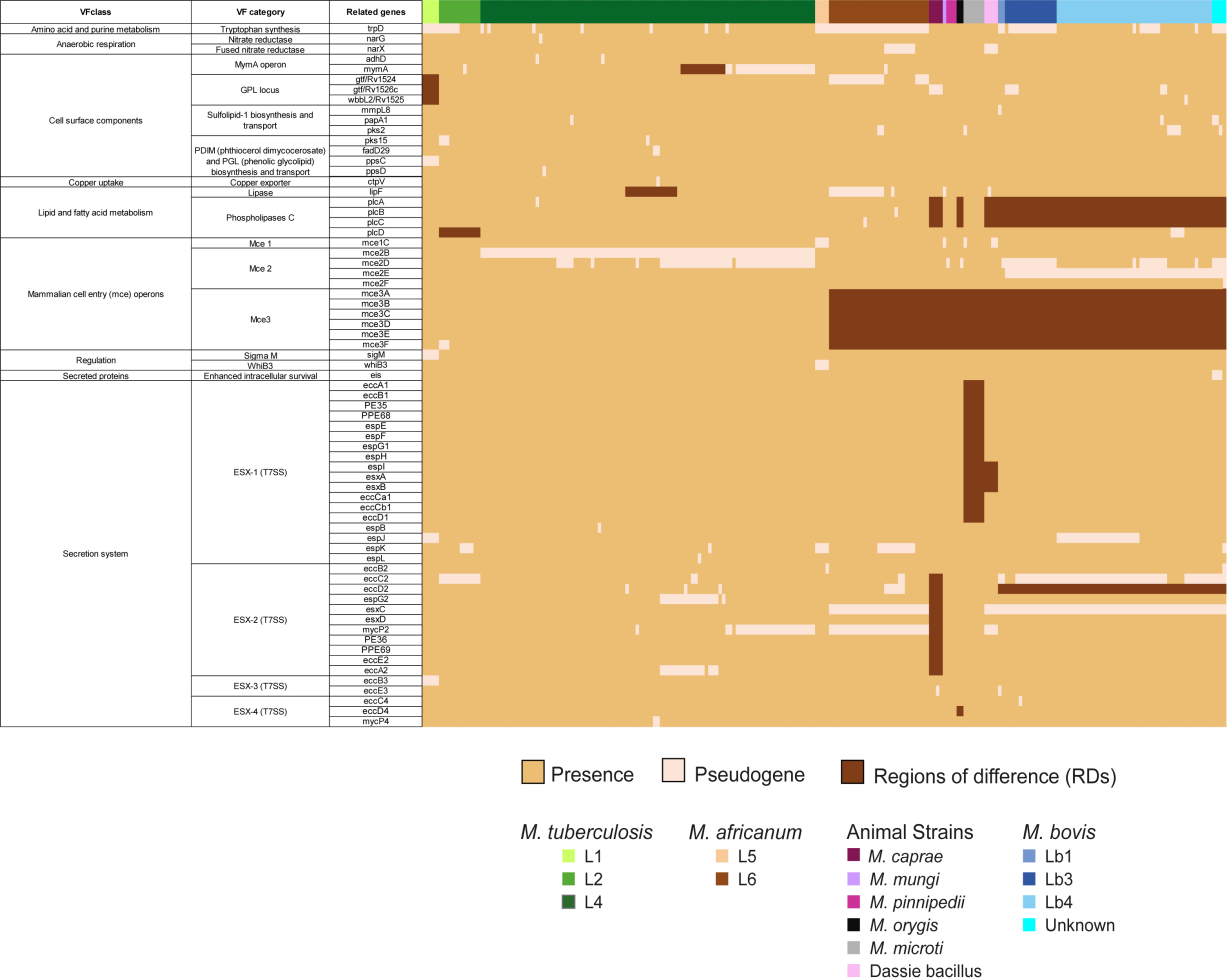
Heatmap of presence and absence of VFs in 233 strains of the MTBC. Colored bars represent MTBC lineages and species. Dark beige indicates the VF protein is present in the genome, while light beige indicates the VF protein is absent and its gene has been predicted as pseudogene by the PGAP of NCBI. Brown indicates the protein is absent because of a deletion (previously described RDs). Genome representatives of *M. tuberculosis* L3, L7, and L8 and *M. africanum* L9 were not detected in the data set, hence not included in the study.

Out of the 227 VFs, 69 were found variably present in the MTBC genomes (Fig. 7; Table S3). These VFs were not universally present due to deletions (RDs) or pseudogenization. Forty-four (63.77%) VFs were prone to pseudogenization; 12 VFs occurred as pseudogenes in only 1 genome, while the remaining 32 pseudogenized VFs were found in 2 to 99 genomes of the data set (Table S3). This indicates that pseudogenization events do not occur uniformly in entire species or lineages, but vary significantly within each bacterial group, as described (29). Importantly, pseudogene herein is defined as a CDS disrupted by a frameshift, non-sense mutation, or insertion of a transposable element as annotated by PGAP. It cannot be definitively stated that these genes are permanently inactivated and non-functional (29). There is always a possibility for neofunctionalization and reversal of frameshift and non-sense mutations (29). This variable gene disruption in the MTBC makes it difficult to predict the phenotype of each ecotype by solely examining the function of individual genes in one bacterial isolate. In addition, among those variably present and/or rarely pseudogenized genes, it is not possible to determine if they emerged independently in each strain or if they occur in monophyletic groups or sublineages.

In agreement with the genome erosion, an accentuated loss of VFs was seen in non-*M*. *tuberculosis* strains (Fig. 7). Nevertheless, hotspots of pseudogenization were detected in *M. tuberculosis: trpD*, *mymA*, GPL (glycopeptidolipids) locus, *ppsC*, *lipF*, *plcD*, *mce2B-D*, *sigM*, and *esx-2* genes were pseudogenized (Fig. 7).

We confirmed our previous observation (29) that *narX* (Rv1736c) is mostly pseudogenized in *M. africanum* L6 genomes when *lipF* (Rv3487c) or *gtf3* (Rv1524) are intact, and vice versa (Fig. 7; Table S3). This pattern is not observed in any other MTBC species. However, the *gtf3* gene, a member of the GPL locus, in addition to *wbbL2* (Rv1525) and *gtf* (Rv1526c), is part of the RD147C of *M. tuberculosis* L1 strains, and *gtf* (Rv1526c) is also pseudogenized in *M. caprae* strains. The *narx* gene is pseudogenized in “dassie bacillus” (Fig. 7; Table S3).

Genes of the biosynthesis of PDIM (phthiocerol dimycocerosate) (*ppsC*, *ppsD,* and *pks15*) appeared pseudogenized in just few *M. tuberculosis* strains (Fig. 7; Table S3). Mutations in these genes have been linked to the inability to synthesize PDIM (84). Studies indicate that *M. tuberculosis* strains may exhibit variations in the composition of PDIM and phenolic glycolipids in their cell walls, which directly impacts their virulence in macrophage and in murine models (85). Furthermore, the pseudogenization of genes of the sulfolipid metabolism (*mmpL8*, *papA1*, *pks2*) was more prevalent in non-*M*. *tuberculosis* strains, suggesting the presence of specific selective pressures in animal strains and *M. africanum* that lead to the erosion of these genes (29). Therefore, the modulation of gene content through pseudogenization in the PDIM biosynthesis genes, the GPL locus, and the sulfolipid biosynthesis genes may contribute to the diversification of cell wall lipid composition among different strains of the MTBC.

The *plcABC* locus is deleted (RD5) in *M. caprae*, *M. orygis*, “dassie bacillus,” and *M. bovis* (75), and it contrasts with the RD152 that included only the *plcD* (Rv1755c) in *M. tuberculosis* L2 (Fig. 7; Table S3). Regulation proteins Whib3 and SigM were pseudogenized in *M. africanum* L5 (29) and *M. tuberculosis* L1, respectively (Fig. 7; Table S3). The redox sensor WhiB3 (Rv3416) (86) is part of the response against reactive oxygen and nitrogen species; a *M. tuberculosis* Δ*whiB3* is highly sensitive to these molecules. While this may help explain the attenuated phenotype of *M. africanum* L5 (86), more genomes should be analyzed to confirm if the *whib3* pseudogenization is present in all L5 strains. SigM (Rv3911), pseudogenized in *M. tuberculosis* L1, has been shown to regulate genes of the synthesis of secreted or surface molecules, including genes of PDIM, ESX-4 secretion system, mycolic acid biosynthesis, and the CpnT exotoxin (87, 88). Additional genomes of *M. tuberculosis* L1 should be inspected to confirm if the *sigM* pseudogenization is conserved among strains.

The *mce* genes, mainly *mce2* and *mce3*, and the *esx* genes, mainly *esx-1* and *esx-2*, were identified as hotspots of gene loss in the MTBC (Fig. 7; Table S3). The *mce1C* (Rv0171) is pseudogenized in all four genomes of *M. africanum* L5, in the *M. mungi* genome, in one genome of *M. microti,* and in two genomes of “dassie bacillus.” While it is uncertain if this pseudogenization occurs in all genomes of these ecotypes, strains of *M. tuberculosis* with a disrupted *mce1* operon are hypervirulent in mice (89) and accumulate free mycolic acid in the cell wall (90). Pseudogenization of at least one gene of the *mce2* operon was detected in few or all *M. tuberculosis* L4, *M. orygis*, *M. pinnipedii,* and *M. bovis* (Fig. 7; Table S3). When knocking out the entire *mce2* operon, a strain of *M. tuberculosis* accumulated sulfolipids in its cell wall (91). The effect of the selected pseudogenization of genes of these operons cannot be predicted. The *mce3* operon, whose function is unknown, is deleted (RD7) in all animal strains and *M. africanum* L6.

ESX-1 genes were found absent due to the known deletions RD1^mic^ and RD1^das^ (92) in *M. microti* and “dassie bacillus,” respectively (Fig. 7; Table S3). In addition, the *espJ* (ESX-1) is pseudogenized in *M. tuberculosis* L1 and many *M. bovis* strains, while the *espK* (ESX-1) is pseudogenized in few *M. tuberculosis* L2 and L4 strains, in *M. africanum* L5, and in many *M. africanum* L6 and *M. bovis* strains (Fig. 7; Table S3). The ESX-1 system secretes proteins that target the host cell membrane, specifically those involved in phagosomal rupture (93).

The pseudogenization of chaperones *eccA2* (Rv3884c) and/or *espG2* (Rv3889c) (93) in few *M. tuberculosis* L4 (Fig. 7; Table S3) suggests secretion modulation of PE/PPE and EspA/C proteins by the ESX-2 system. The *esxC* and *mycP2* genes are also significantly pseudogenized in the data set; *esxC* (Rv3890c) is pseudogenized in *M. africanum* L6, “dassie bacillus,” and *M. bovis*; and *mycP2* (Rv3886c) is pseudogenized in few *M. tuberculosis* L4, *M. africanum* L6, and in “dassie bacillus.” Gene deletion within ESX-2 is observed in other strains as well. For instance, most genes are deleted in *M. caprae* (RDcap_Spain7), and *eccD2* is deleted in *M. bovis* (RDbovis) (Fig. 7; Table S3). Additionally, a deletion encompassing the ESX-2 operon, referred to as RDpan, occurs in pyrazinamide-sensitive lineages of *M. bovis* (94) (not included in the data set). The ESX-2 system has been reported as not required for *in vitro* growth or virulence of *M. tuberculosis* in mice (86). Finally, in ESX-4, *eccD4* (Rv3448) is part of the RD315 in *M. orygis*, while *mycP4* (Rv3449) is pseudogenized in two *M. tuberculosis* L4 strains (Fig. 7; Table S3).

Taken together, the modulation of VFs suggests that virulence in MTBC strains is a multifactorial and complex process, involving the disruption or deletion of many genes simultaneously. In addition, gene disruptions of VFs, represented by pseudogene annotations, are mostly not conserved between different species, being poor predictors of MTBC ecotypes. Their heterogeneous disruptions within MTBC ecotypes suggest that this may be a mechanism of host immune system evasion for each ecotype, particularly in the case of phase variation. For instance, it is possible that the different ecotypes use phase variation, turning genes on and off to modulate their interaction with the host. In fact, what was observed in these genomic comparisons is just a snapshot of gene sequences that may be subjected to phase variation. One example is the recently described phase variation of the *glpk* (glycerol kinase) gene, which modulates the cell wall composition of *M. tuberculosis* (95).

### Functional capacity of the accessory genomes

To identify additional genes subjected to modulation within each species and that are not in the VFDB, we evaluated the accessory genomes of each bacterial group (Table 2) with STRING. A total of 18.8% (47/250), 33.8% (52/154), 26.0% (33/127), and 5.8% (9/155) groups of orthologous proteins of the accessory genome of *M. tuberculosis* (G1), *M. africanum* (G2), *M. bovis* (G3), and “other animal strains” (G4), respectively, were annotated as hypothetical proteins. Gene IDs of the remaining proteins were identified with STRING using *M. tuberculosis* H37Rv as reference. Only three proteins (*eccD2*, *mycP2*, *espK*) with gene ID were common among all bacterial groups, suggesting that the targets of gene loss are not the same for all species/ecotypes (Fig. S7).

In addition to the VFs described above, other important proteins appear in the accessory genome of each bacterial group. Accordingly, seven main protein networks were identified in *M. tuberculosis* (Fig. S8): lipid metabolism (PDIM: *ppsD*, *ppsC*, *fadD29;* sulfolipids: *papA1*; phospholipids: *plsB1*; triacylglycerol: *tgs3*; mycolic acids: *pks13; bioF2*, among others), proteins of the ESX-1 (*espB*, *espk*, *espJ*, *espL*, PE35); proteins of ESX-2 (*mycP2*, *espG2*, *eccA2*, *eccD2*), two networks of mobile elements (network 1: Rv1572c-Rv1574, Rv1576-Rv1586c; network 2: Rv2651c-Rv2658c), proteins associated with the serine/threonine kinase PknK (*pknK*, *moeY*, *moaA1*, *kdpD*, Rv1973), and sigma factors SigM and SigL.

In *M. africanum*, seven protein networks were identified (Fig. S8): Mce1 and 3 (*mce3A-F*, *yrbE3B*, *mce1C*); biosynthesis of molybdenum cofactor (*moaA1*, *moaC1*, *moaC3*, *narX*); lipid metabolism (sulfolipids: *pks2*; diacyltrehaloses/polyacyltrehaloses: *pks3*, *pks4*, *fadD21*; other polyketide synthases: *pks7*, *pks8*, *pks10*, *pks12; lipF*, *lipM*); phospholipases (*plcA-C*); proteins of ESX-2 (*esxN*, *mycP2*, *esxC*, *eccD2*, *eccC2*, *eccB2*); metal homeostasis (*mymT*, *cmtR*, *ctpJ*); and proteins associated with the serine/threonine kinase PknH (*embR*, *mec*, *echA1*).

In *M. bovis*, five protein networks were identified (Fig. S9): lipid metabolism (PDIM: *ppsA*, *ppsB*, *ppsD*; sulfolipids: *papA1*, *pks2*, *mmpL8*; mycolic acids: *pks13*; other polyketide synthases: *pks12*, *pks7*; *bioF2*, among others); Mce2 (*mce2D*-*F*); carbon metabolism (*glpk*, *xylB*, *rpe*, *cya*); proteins of ESX-1 and ESX-2 (*espK*, *espJ*, *espB*, *eccD2*, *mycP2*); and DNA repair (*alkA*, *dnaQ*, *recC*).

In other animal strains, nine protein networks were identified (Fig. S9): cobalamin synthesis (*cobK*, *cobN*, *cobI*); lipid metabolism and cytochrome P (PDIM: *ppsC*, *ppsD*; CyP: *cyp141*, *cyp130*, *cyp142*, *cyp121; fadD15*, *fadE18*, *fadE4*, *fadB2*, *fadB5*); proteins of ESX-1 and ESX-2 (*mpt64*, *esxH*, *esxN*, *esxB*, *eccCa1*, *espC*, *esxA*, *espk*, *eccD1*, *esxD*, *mycP2*, *eccD2*, *eccE2*, PPE69, PE36); DNA repair (*recB-C*); Mce1 and 2 (*mce2D*, *yrbE2A*, *mce1C*); breakdown enzymes (*lipJ*, *lipN*, *nlhH*, *amiB2*, Rv2531c, *arcA*, *rocE*); phospholipases (*plcA-C*); proteins associated with the serine/threonine kinase PknH (*mshA*, *rpoB*, *embR*, *lprH*, *glpK*); phosphate transport (*pitB*, *pstB*); and sulfur/cystein metabolism (*cysA2*, *cysN*).

While there were few proteins common to two or more bacterial groups (Fig. S7), the differences observed between species underscore selective pressures unique to each ecological niche. Apart from the known VFs, gene content modulation was not present in central metabolic pathways; instead, it was seen in regulatory proteins (*pknH*, *pknK, oxyR, whiB3*), DNA repair, lipid metabolism, phosphate transport, metal homeostasis, and mobile elements. The loss of regulatory proteins suggests significant changes in metabolic states with substantial impact on phenotype. In addition, the loss of genes of PDIM biosynthesis, except in *M. africanum*, suggests that the phenomenon of PDIM spontaneous loss (96) may be less common in these species or that other types of mutations are responsible for inactivating these genes in *M. africanum*.

### Limitations of this study

Analyzing gene content is difficult because most available genomes are not closed. Closing a genome is problematic for genome assemblers due to short read lengths and repetitive regions (97). Although third-generation sequencing (e.g., PacBio or Oxford Nanopore) has improved genome assembly with longer reads, the high error rate produces false indels that overestimate truncated genes (98). In fact, many *M. tuberculosis* genomes deposited in GenBank assembled using long reads, even in a hybrid form with Illumina reads, have an overestimation of pseudogenes (29). Therefore, genomes (closed or drafts) sequenced with these platforms, and others known to have higher error rates in homopolymeric tracts (e.g., 454 and Ion Torrent), were not included in this study. More details about the errors detected in these genomes can be found in Fig. S10. In contrast, Illumina sequencing has very low error rates for indels (99), which is the main type of error that may interfere with gene prediction. Importantly, despite these limitations, pan-genome studies with draft genomes have yielded important findings for many bacterial species (70, 100–102) and were thus included herein.

It is likely that many closed genomes of *M. tuberculosis* were assembled using H37Rv strain as the reference genome. Thus, the assembly of repetitive regions (where gaps normally happen) may be biased toward the H37Rv sequence. On the other hand, due to the nature of the short reads and assembler limitations, repetitive regions may also carry assembly errors. Therefore, some of the gene variations observed herein may be related to biased or erroneous assembly of repetitive regions such as PE/PPE genes, *pks* genes, transposases, integrases, maturases, phage, and repetitive family 13E12 genes.

The genome data set used herein does not include representatives of all lineages of *M. tuberculosis* and *M. africanum*. Strains of L3, L7, L8, and L9 were not available according to our criteria. L1, L2, and L5 are also underrepresented compared to L4. Therefore, conclusions drawn from this study should consider potential biases associated with *M. tuberculosis* and *M. africanum* lineages. Noteworthy, the focus of our study was to show differences among the MTBC ecotypes. All lineages of *M. tuberculosis* are part of the same ecotype and highly adapted to the human host. Thus, while there may be some differences among lineages of *M. tuberculosis* and *M. africanum*, findings of this study are the first step to better understand the gene variations among the different MTBC ecotypes.

The use of relaxed core genome (95% cutoff) instead of a strict core genome (99%) for pan-genome studies with draft genomes has circumvented some of the limitations described above (103), and was applied herein. In addition, to understand the impact of draft genomes on the pan-genome prediction, correlation graphs were generated between the number of strain-specific proteins of each genome and their number of contigs (Fig. S11A) or N50 (Fig. S11B). No significant correlations were found between these variables, indicating that the number of strain-specific proteins is not a consequence of genome assembly artifacts. Noteworthy, significant genomic erosion was equally observed in the almost-closed genomes of *M. africanum* (with <10 contigs). Thus, draft genomes likely have a minor influence on the genomic decay detected in this study.

The number of strain-specific proteins was also very low (~10 proteins/strain). Due to the high conservancy of MTBC genomes, the emergence of new genes is not expected. Thus, it is possible that these are mistaken gene predictions on alternative strands, genes predicted in intergenic regions, short gene predictions upstream of a gene with a wrong start site annotation, or misassemble of repetitive areas resulting in inaccurate gene predictions. In MTBC genomic studies, repetitive genes like PE/PPE and mobile genetic elements are commonly excluded. While eliminating these genes was a possibility herein, pan-genome studies are not complete without all genes from a given organism. Thus, we opted to maintain them.

### Final considerations

Our study revealed that members of the MTBC have significant variations in their gene repertoire due to gene loss, not only because of RDs, but also because of gene truncation/pseudogenization. This is the first study to analyze the impact of gene loss through RDs and pseudogenization/gene truncation simultaneously on the pan-genome of the MTBC. Herein, we extended a prior analysis (29) that focused on pseudogenes and gene truncations of *M. tuberculosis*, *M. africanum,* and *M. bovis* to encompass the majority of the MTBC ecotypes, and within the framework of its pan-genome. It is important to highlight that we may be observing snapshots of certain genes that are subjected to phase variation through frameshift, hence their loss is not definitive in a particular strain or even ecotype. This reflects how these pathogens are able to vary their genetic makeup in the absence of horizontal gene transfer.

Diverse patterns of gene erosion among MTBC species and lineages were observed, suggesting that their gene repertoire is shaped by unique selective pressures experienced by each one of these species or lineages. Additional evidence further supported the phenomenon of differential gene selection by ecotype. First, a significant drop in the number of shared-genes families between genomes was seen only when inter-species comparisons were made (Fig. 3B), suggesting that species-specific ecological characteristics are associated with changes in gene content. Second, the gene repertoire of *M. africanum* L6 was shaped throughout evolution in a manner that was not expected based on its SNP-based phylogeny. As gene repertoire variations in the MTBC are driven by gene loss through deletions and frameshifts, they may not accurately reflect the SNP-based phylogeny. Third, gene loss affects functional classes and virulence factors differently across MTBC species, and each bacterial group has its unique accessory genome. Therefore, while variations in the pan-genome of the MTBC seem small compared to other non-clonal bacteria, the changes in their gene content are significant enough to generate phenotypes that challenge the control of the disease. Additionally, our study also clarified discrepancies in the literature about the MTBC pan-genome, showing it to be closed when calculated using high-quality genome annotation.

During host-to-host transmission, MTBC undergoes population bottlenecks that contribute to the relaxation of natural selection and increase in genetic drift (83). Herein, strains of *M. tuberculosis* L4 (and possibly *M. tuberculosis* L1) were the least affected by gene loss. While the increase in genetic drift has been used to explain the genomic erosion in the MTBC (29, 49), it is also important to understand the mechanisms by which genotypic heterogeneity emerges more frequently in non-L4 strains compared to L4. Targeting such mechanisms may help fight the emergence of virulent ecotypes or sublineages, as well as antibiotic resistance.

We also show that the genomic erosion in MTBC is a variable and active process with potential effects on virulence. The consequences of genomic erosion on phenotype require experimental evaluation, as gene loss may not always lead to attenuation (104, 105) but could instead provide adaptive traits for these bacteria to survive in new environmental niches or evade the host immune system. The diversity of gene repertoire within species and lineages challenges the determination of species or lineage-specific phenotypes, cautioning against generalizations based on laboratory observations with single genes and isolates.

## Data Availability

All scripts used in this study are available at GitHub.

## SUPPLEMENTAL MATERIAL

Supplementary figures spectrum.02289-23-s0001.pdf Figures S1 to S11.

Supplementary file 1 spectrum.02289-23-s0002.pdf Results from COG analyses of bacterial groups G1-G4.

Table S1 spectrum.02289-23-s0003.xlsx Genome dataset.

Table S2 spectrum.02289-23-s0004.xlsx Patristic distance and %GRR of the MTBC genomes

Table S3 spectrum.02289-23-s0005.xlsx Virulence factors of the MTBC.

ASM does not own the copyrights to Supplemental Material that may be linked to, or accessed through, an article. The authors have granted ASM a non-exclusive, world-wide license to publish the Supplemental Material files. Please contact the corresponding author directly for reuse.
