# Supplementary material for: Diversification of gene content in the Mycobacterium tuberculosis complex is determined by phylogenetic and ecological signatures
Source: Microbiol Spectr. 2024 Jan 17;12(2):e02289-23. doi: 10.1128/spectrum.02289-23 (PMC10871547; doi:10.1128/spectrum.02289-23)
Supplement: Supplementary figures — Figures S1 to S11. [file spectrum.02289-23-s0001.pdf]

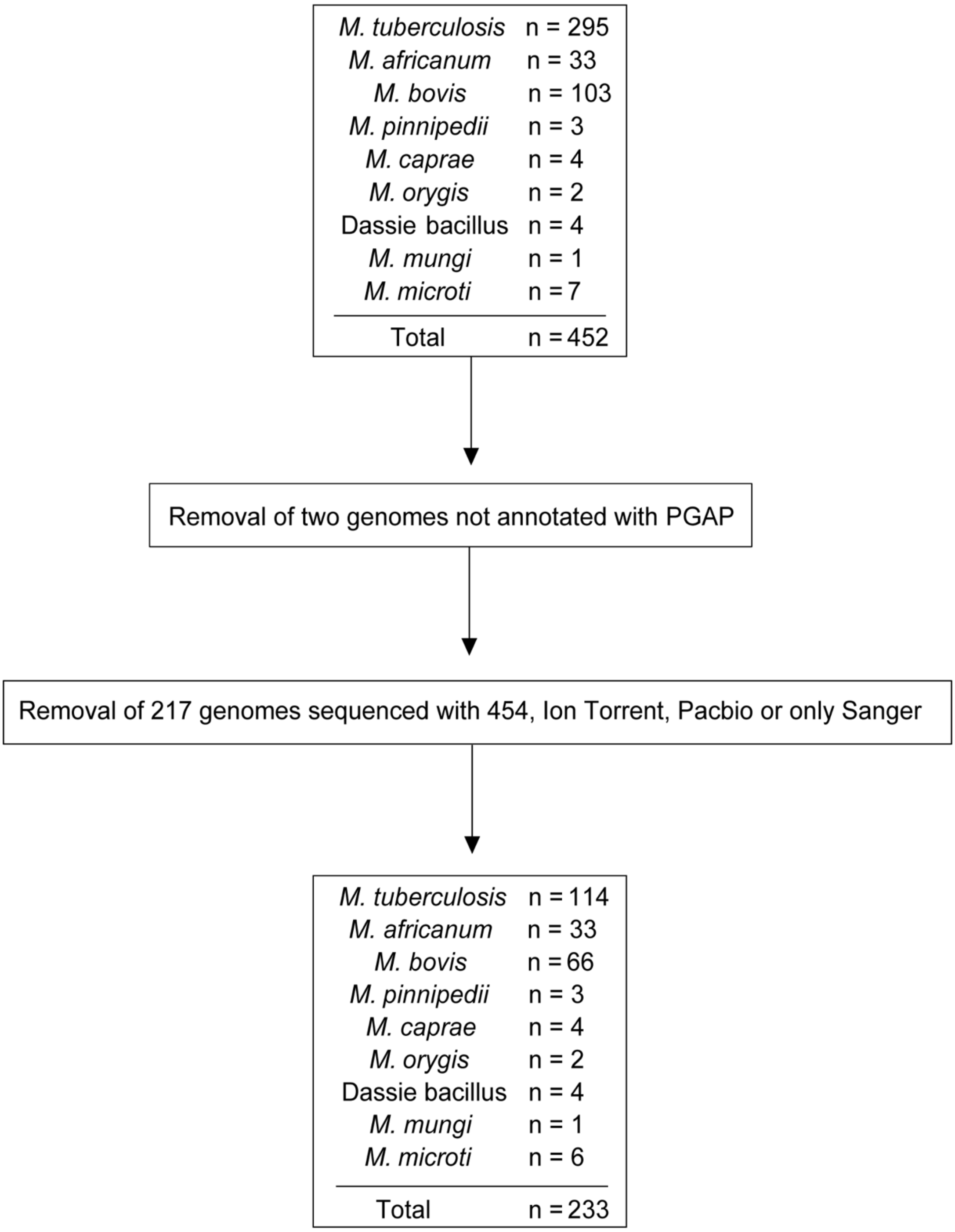

Figure S1. Flowchart of the curation performed during the selection of *Mycobacterium tuberculosis* complex genomes deposited at RefSeq, National Center for Biotechnology Information (NCBI) in September 2022.

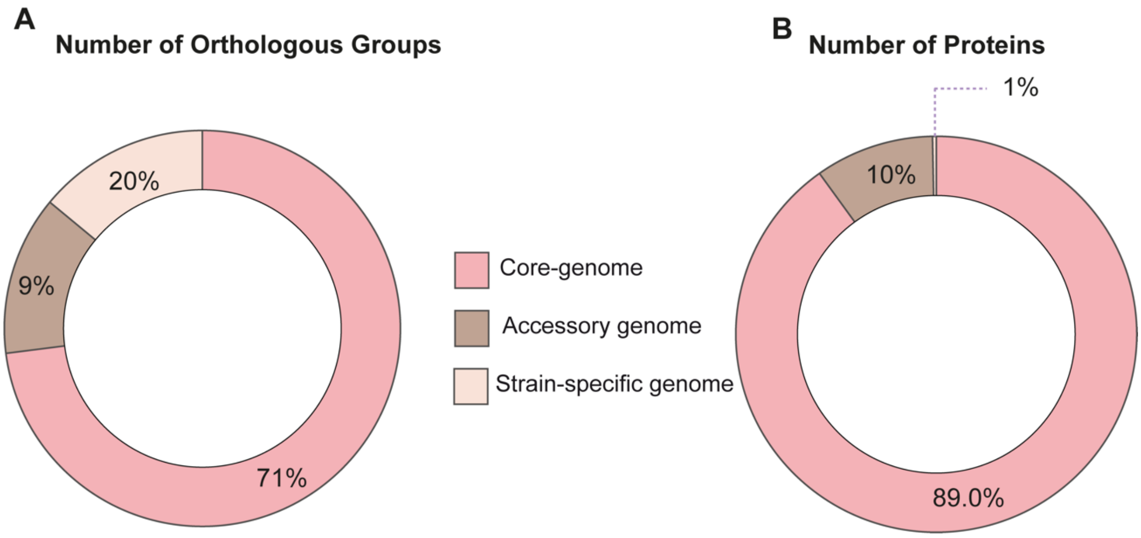

Figure S2. Pie charts representing the percentage of the number of orthologous protein clusters (**A**) and proteins (**B**) predicted from genes and pseudogenes of the *Mycobacterium tuberculosis* complex (MTBC) genomes.

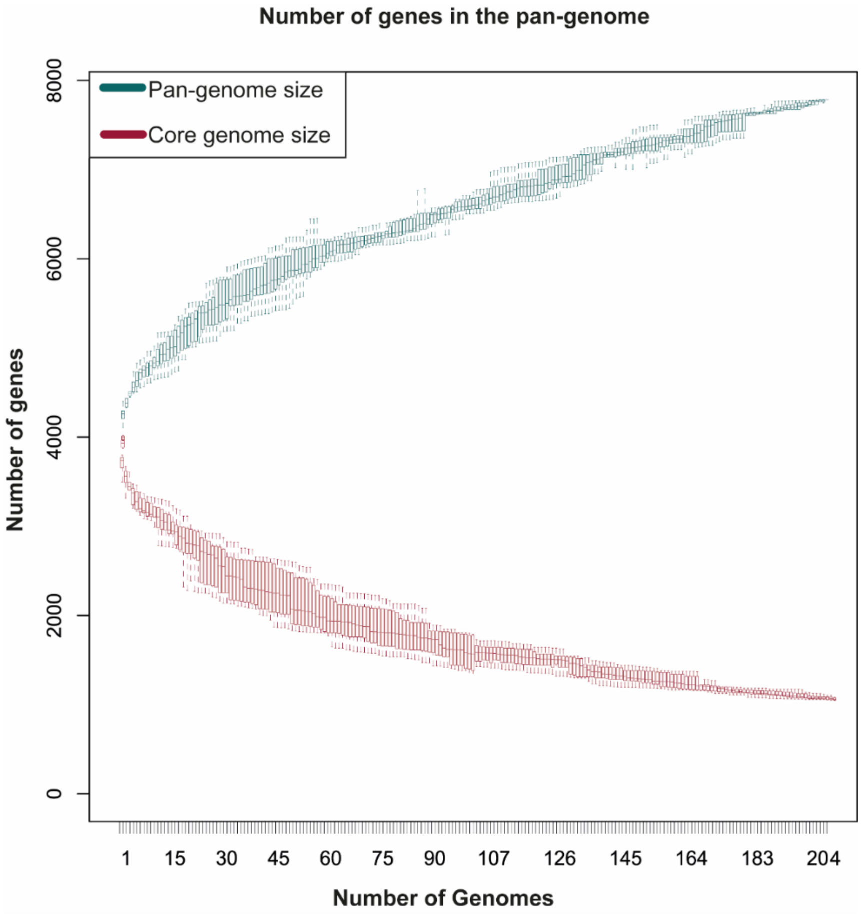

Figure S3. Orthologous protein clusters of the pan-genome detected by the sequential addition of genomes of the *Mycobacterium tuberculosis* complex (MTBC). Each boxplot represents the distribution of the number of orthologous protein clusters from 100 randomly generated strain orders. The refined power-law pan-genome model [1] was applied using the *panmatrix* and *heaps* functions of micropan R package [2]. Calculated alpha parameter = 1.5 If the exponent alpha > 1, that bacterial group has a closed pan-genome.

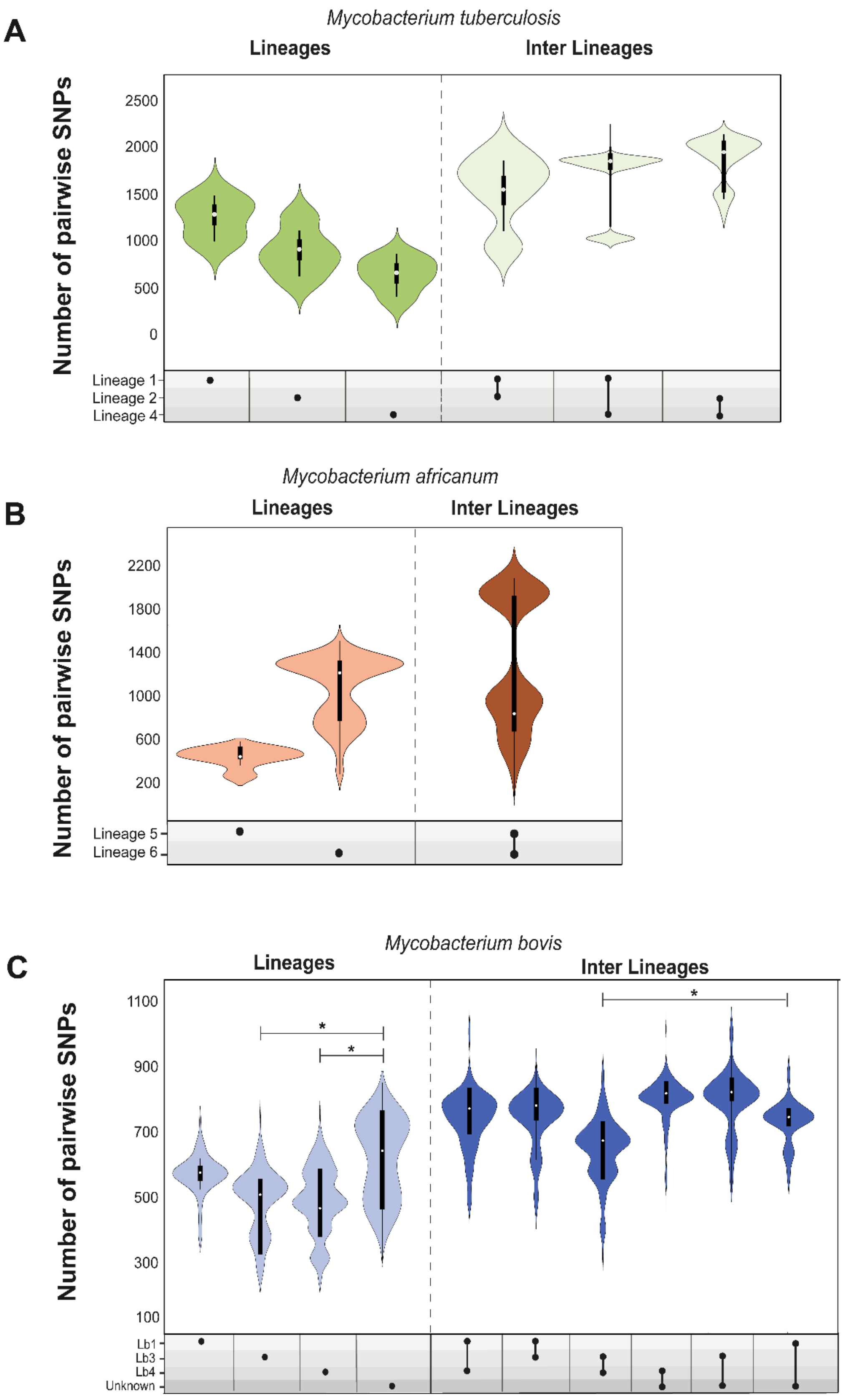

Figure S4. Pairwise single nucleotide polymorphism (SNP)-distance between genomes of species of the *Mycobacterium tuberculosis* complex (MTBC) according to lineages. (**A**) *Mycobacterium tuberculosis,* (**B**) *Mycobacterium africanum*, (**C**) *Mycobacterium bovis*.

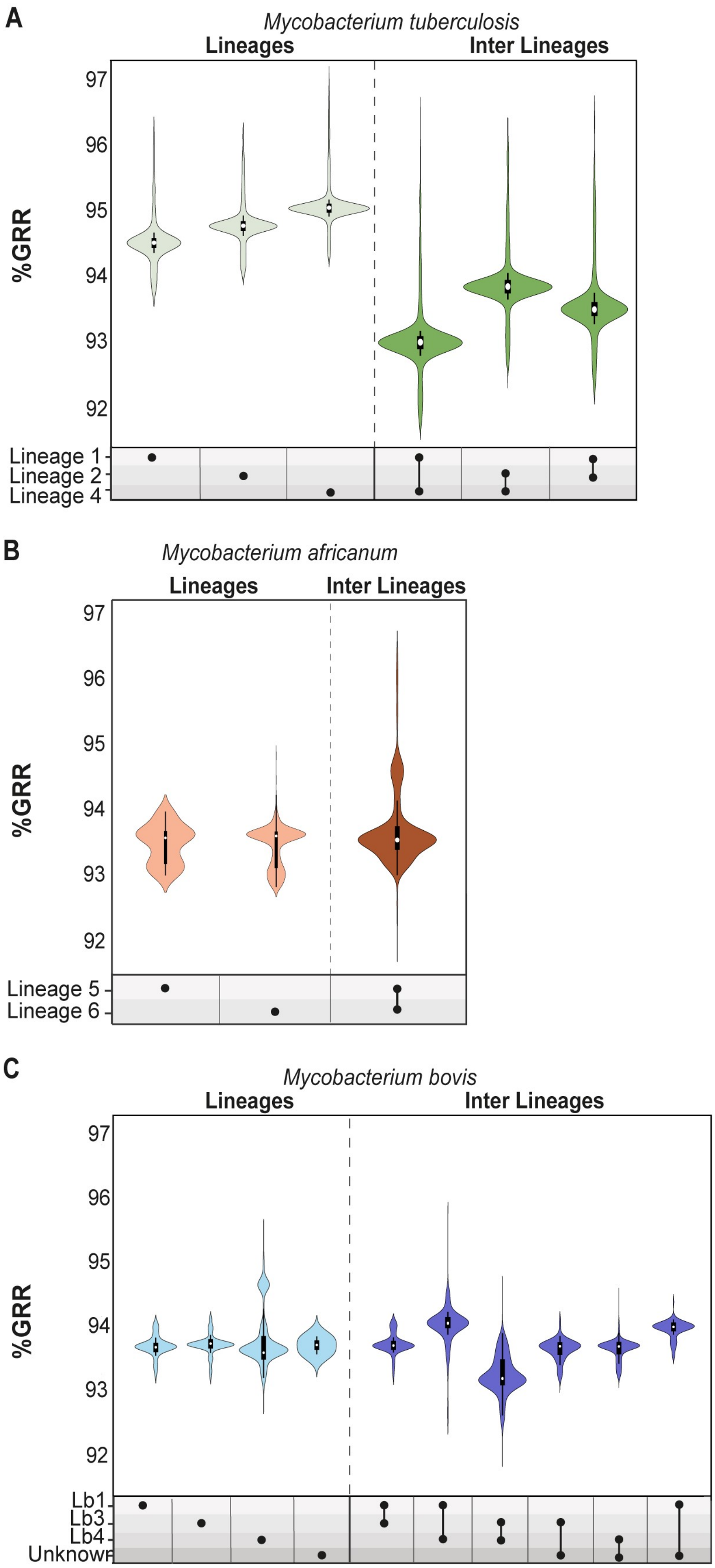

Figure S5. Gene repertoire relatedness (GRR) between genomes of species of the *Mycobacterium tuberculosis* complex (MTBC) according to lineages. (**A**) *Mycobacterium tuberculosis,* (**B**) *Mycobacterium africanum*, (**C**) *Mycobacterium bovis*.

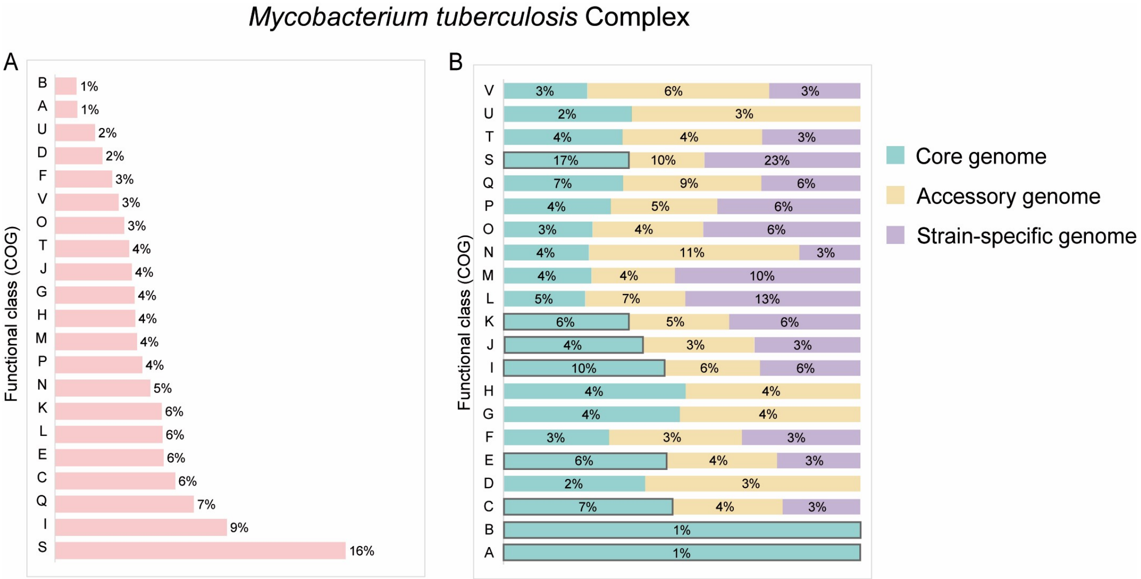

Figure S6. Cluster of Orthologous Groups (COG) of the *Mycobacterium tuberculosis* complex (MTBC) and enrichment analysis. **(A)** Functional classification of the COG of the proteome of 233 genomes of the MTBC. **(B)** Functional classification of the COG of the MTBC according to the pan-genome. Axis x: Percentage of proteins of the MTBC. Axis y: COG Functional Classification. COG categories are: [D] Cell cycle control, cell division, chromosome partitioning; [M] Cell wall/membrane/envelope biogenesis; [N] Cell motility; [O] Post-translational modification, protein turnover, and chaperones; [T] Signal transduction mechanisms; [U] Intracellular trafficking, secretion, and vesicular transport; [V] Defense mechanisms; [W] Extracellular structures; [Y] Nuclear structure; [Z] Cytoskeleton; [A] RNA processing and modification; [B] Chromatin structure and dynamics; [J] Translation, ribosomal structure and biogenesis; [K] Transcription; [L] Replication, recombination and repair; [C] Energy production and conversion; [E] Amino acid transport and metabolism; [F] Nucleotide transport and metabolism; [G] Carbohydrate transport and metabolism; [H] Coenzyme transport and metabolism; [I] Lipid transport and metabolism; [P] Inorganic ion transport and metabolism; [Q] Secondary metabolites biosynthesis, transport, and catabolism; [R] General function prediction only; [S] Function unknown; [X] Mobilome components. More than 89% of the CDS (coding DNA sequences) of each group were successfully annotated with EggNOG. Categories highlighted with bold edges were significantly enriched ($p<0.05$) compared to the whole pan-genome.

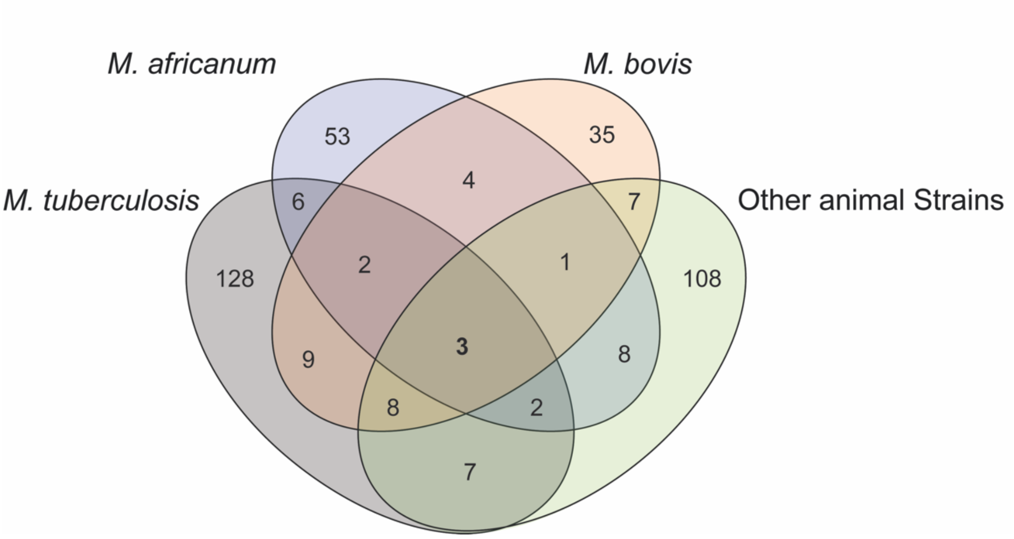

Figure S7. Venn diagram of shared proteins of the accessory genome of each bacterial group. The groups of orthologous proteins of the accessory genomes (except the hypothetical clusters) of each bacterial group were evaluated using STRING [3] using *Mycobacterium tuberculosis* H37Rv as reference. The list of gene IDs identified by STRING for each bacterial group was matched to generate this Venn Diagram. Thus, only proteins with a gene ID as detected by STRING are included.

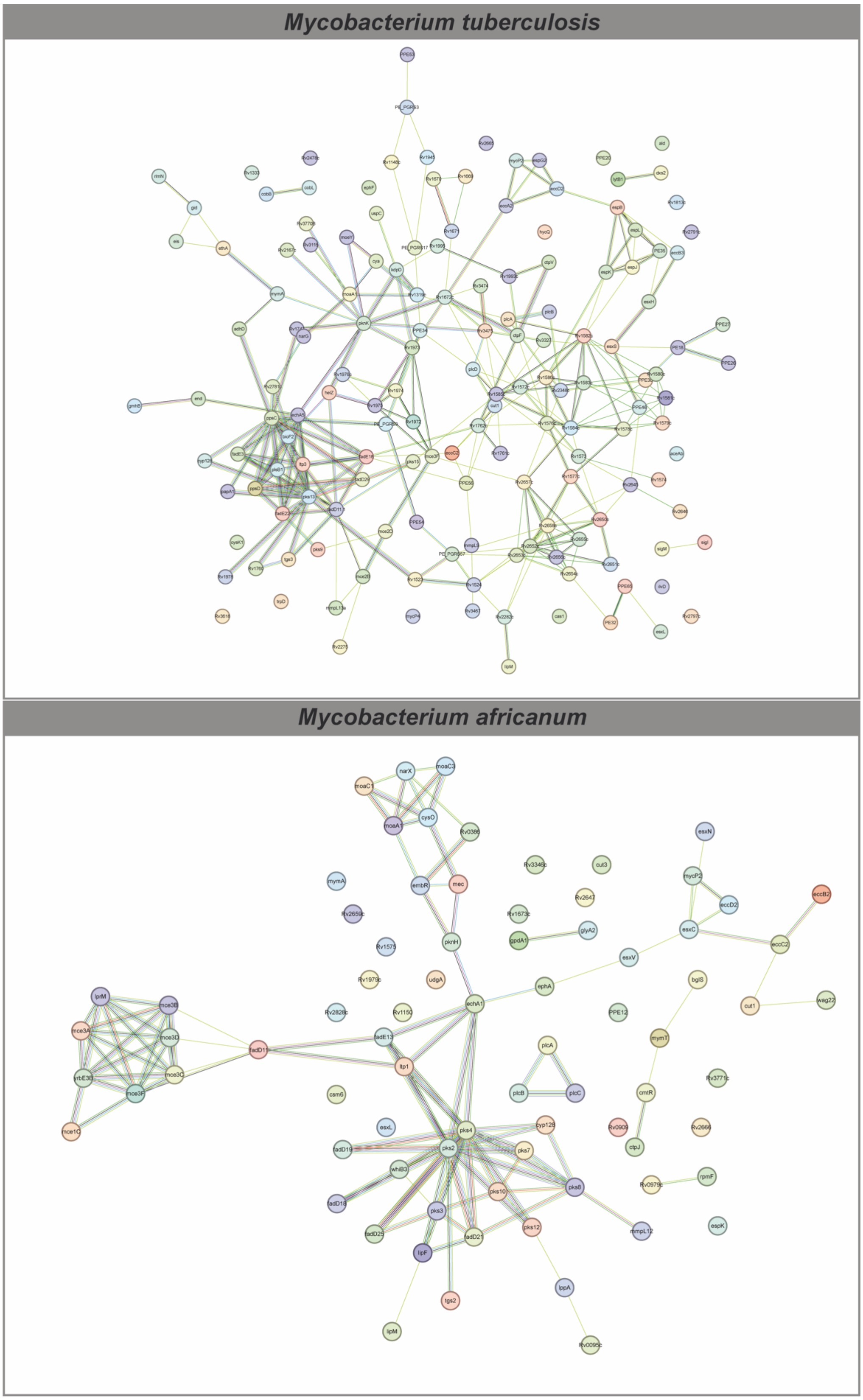

Figure S8. Protein networks of the accessory genomes of *Mycobacterium tuberculosis* and *Mycobacterium africanum*. The groups of orthologous proteins of the accessory genomes (except the hypothetical clusters) of each bacterial group were evaluated using STRING [3] using *Mycobacterium tuberculosis* H37Rv as reference. Edges - aqua green: from curated databases; pink: experimentally determined; green: gene neighborhood; yellow: text-mining; black: co-expression.

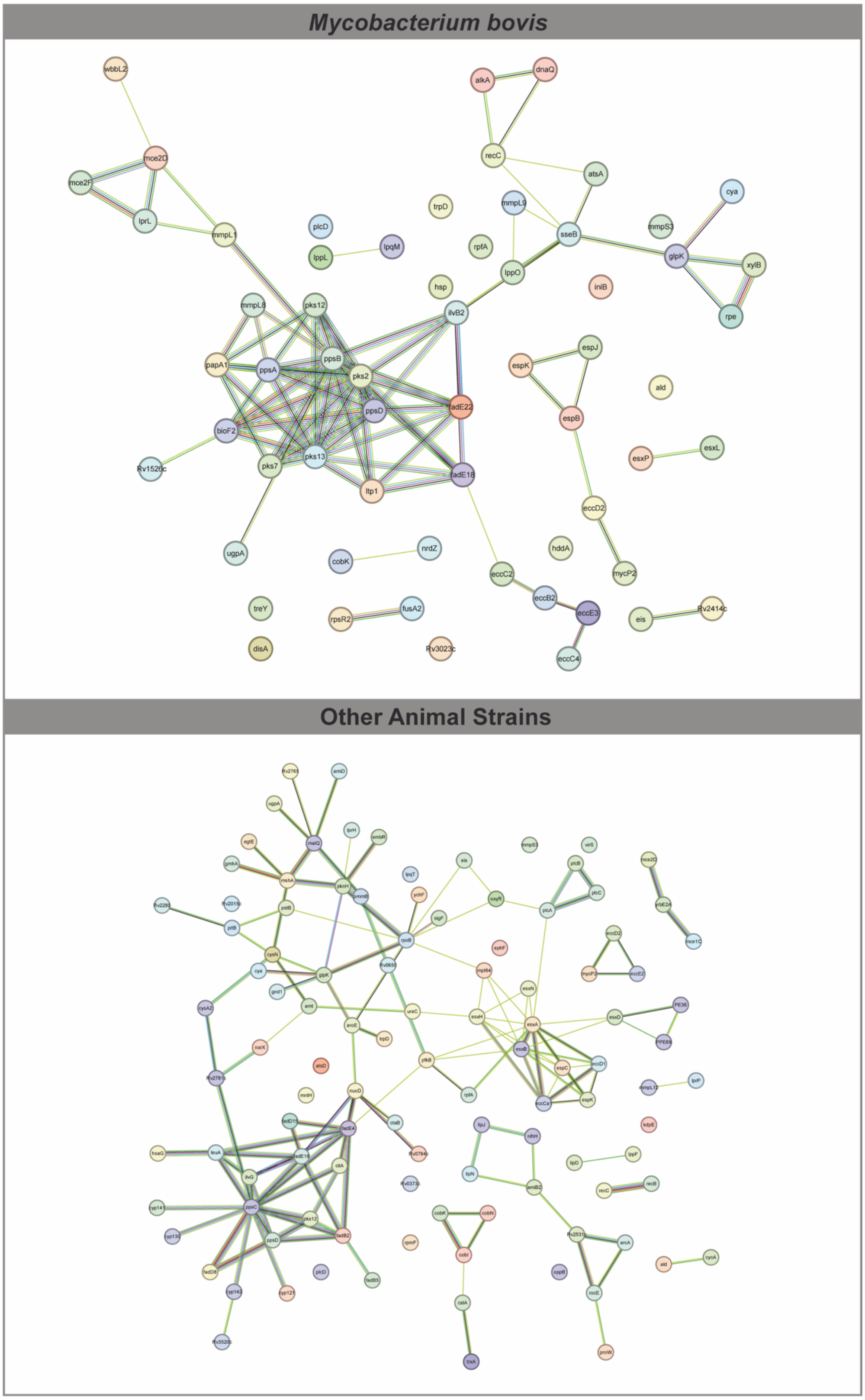

Figure S9. Protein networks of the accessory genomes of *Mycobacterium bovis* and 'other animal strains'. The groups of orthologous proteins of the accessory genomes (except the hypothetical clusters) of each bacterial group were evaluated using STRING [3] using *Mycobacterium tuberculosis* H37Rv as reference. Edges - aqua green: from curated databases; pink: experimentally determined; green: gene neighborhood; yellow: text-mining; black: co-expression.

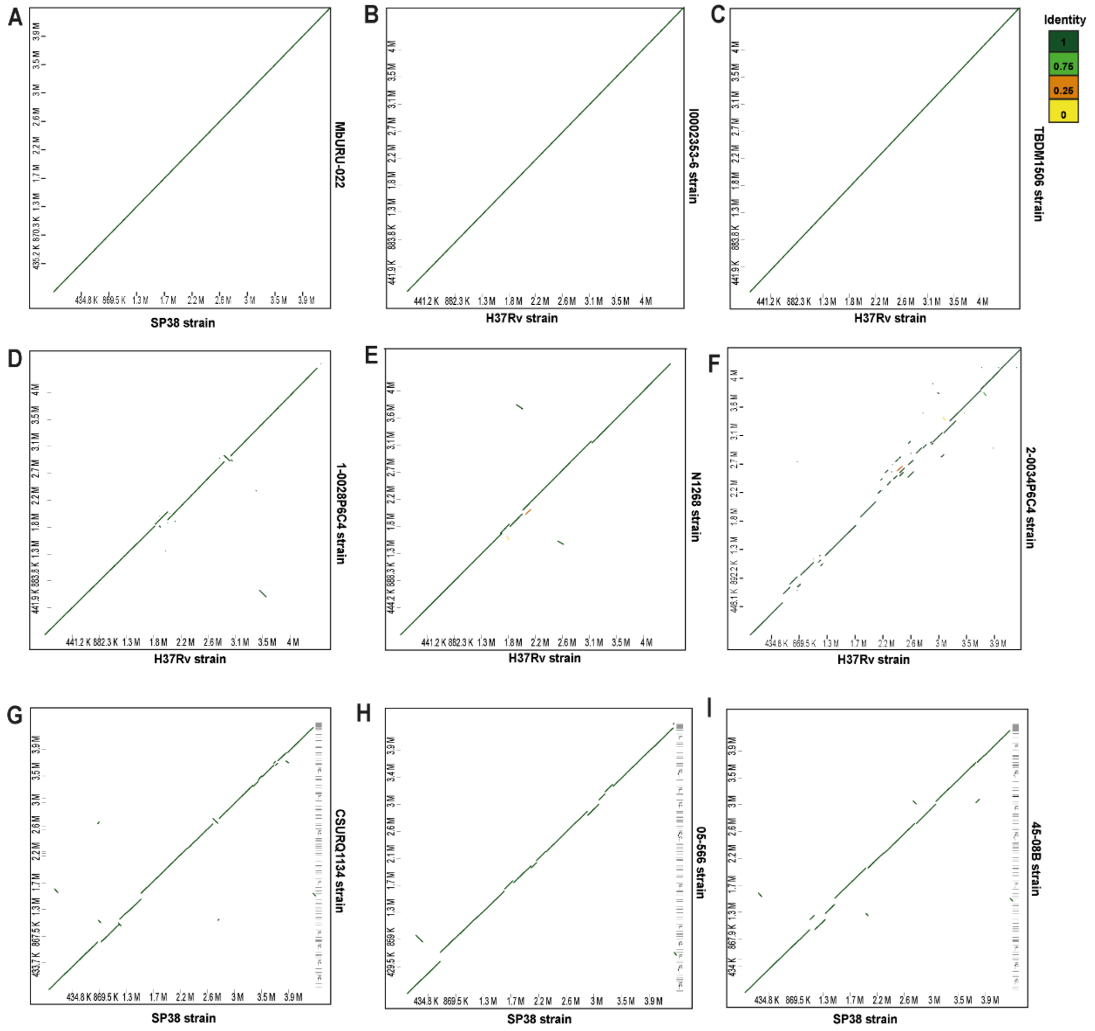

Figure S10. Dotplot comparisons of good-quality and low-quality genomes of *Mycobacterium tuberculosis* or *Mycobacterium bovis* against the reference genome sequences of *M. tuberculosis* H37Rv or *M. bovis* SP38. The reference genomic sequence is on the x-axis; sequences of the other strains are on the y-axis. **A-C** are examples of quality-approved genomes. **D-I** are the examples of genomes sequencing by PacBio or IonTorrent plataform that did not pass quality control. Dotplots were generated R package *seqinr*.

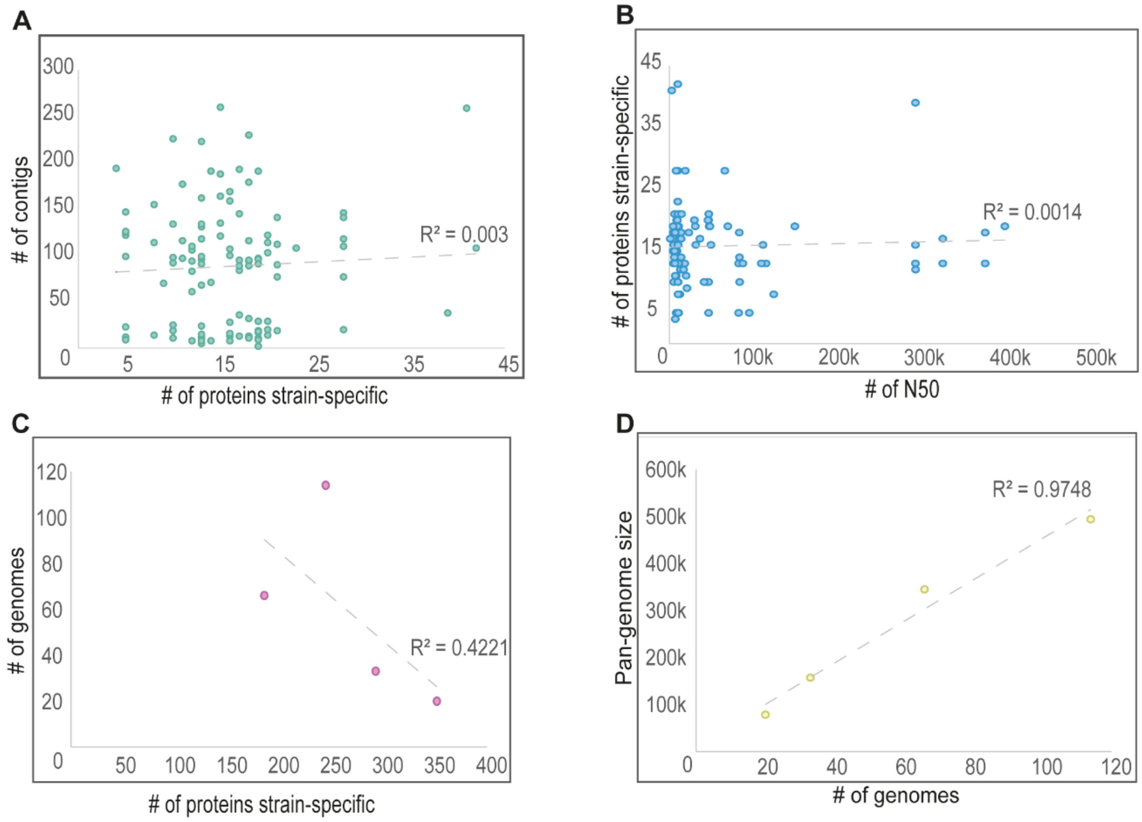

Figure 11. Correlation graphs of 233 strains of the *Mycobacterium tuberculosis* complex. (**A**) Correlation between the number of strain-specific proteins and the number of contigs in each draft genome. (**B**) Correlation between the number strain-specific proteins and the N50 of each draft genome. (**C**) Correlation between the number strain-specific proteins and the number genomes of *M. tuberculosis* (n=114), *M. africanum* (n=33), *M. bovis* (n=66) and animal strains (n=20) (each dot corresponds to one group). (**D**) Correlation between the pan-genome size and number of genomes of of *M. tuberculosis* (n=114), *M. africanum* (n=33), *M. bovis* (n=66) and animal strains (n=20) (each dot corresponds to one group).

References

1. Tettelin, H.; Riley, D.; Cattuto, C.; Medini, D. Comparative genomics: the bacterial pan-genome. *Curr. Opin. Microbiol.* **2008**, *11*, 472–477.
2. Snipen, L.; Liland, K.H. micropan: An R-package for microbial pan-genomics. *BMC Bioinformatics* **2015**, *16*, 1–8.
3. Jensen, L.J.; Kuhn, M.; Stark, M.; Chaffron, S.; Creevey, C.; Muller, J.; Doerks, T.; Julien, P.; Roth, A.; Simonovic, M.; et al. STRING 8—a global view on proteins and their functional interactions in 630 organisms. *Nucleic Acids Res.* **2009**, *37*, D412–D416.
