# Supplementary material for: Diversification of gene content in the Mycobacterium tuberculosis complex is determined by phylogenetic and ecological signatures
Source: Microbiol Spectr. 2024 Jan 17;12(2):e02289-23. doi: 10.1128/spectrum.02289-23 (PMC10871547; doi:10.1128/spectrum.02289-23)
Supplement: Supplementary file 1 — Results from COG analyses of bacterial groups G1-G4. [file spectrum.02289-23-s0002.pdf]

Silva-Pereira et al. Diversification of gene content in the *Mycobacterium tuberculosis* complex is determined by phylogenetic and ecological signatures.

Supplementary file 1.

COG analyses of bacterial groups (G1-G4)

When analyzing the bacterial groups separately (G1-G4), most COG categories were represented by core, accessory, and strain-specific proteins of each bacterial group. However, the nucleotide transport and metabolism (F) category was detected in the accessory genomes of all groups, except in *M. tuberculosis* (G1) (Figure 1). This finding is explained by the pseudogenization of the *trpD* gene (Rv3487c, anthranilate phosphoribosyltransferase) in 52% of the non-*M. tuberculosis* genomes analyzed herein. However, because the mutation occurs at the last 3’ six amino acids of the TrpD, this protein is likely functional and its annotation as a pseudogene should be reviewed [1]. Similarly, the translation, ribosomal structure, and biogenesis (J) category was detected in all accessory genomes, except in *M. africanum* (Figure 1). The variable presence of the *rpmB* (50S ribosomal protein L28) and *lprB* (Rv1274; putative lipoprotein LprB) genes in non-*M. africanum* genomes account for this discrepancy.

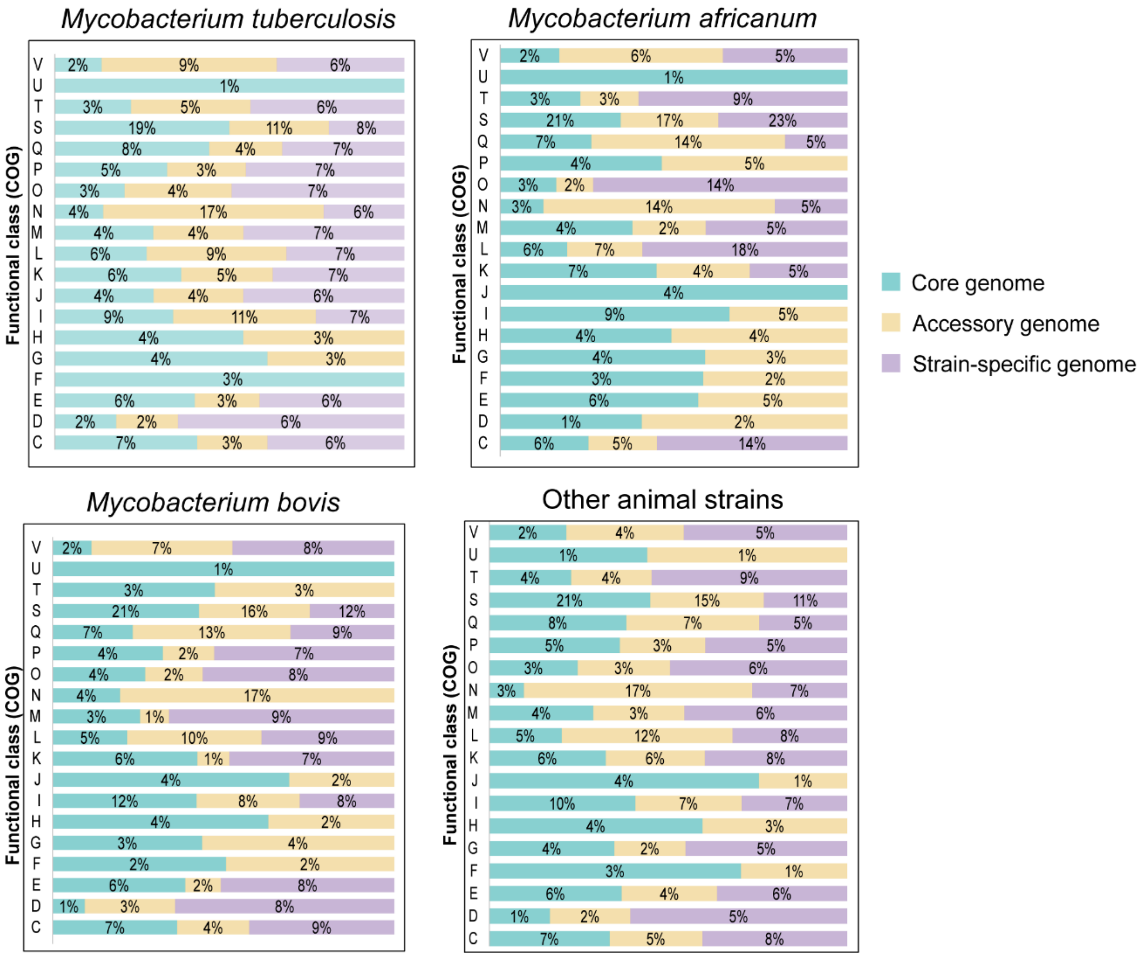

Figure 1. Cluster of Orthologous Groups (COG) of each bacterial group of the *Mycobacterium tuberculosis* complex (MTBC) according to their pan-genome. Axis x: Percentage of proteins of each bacterial group. Axis y: COG Functional Categoryification. COG categories are: [D] Cell cycle control, cell division, chromosome partitioning; [M] Cell wall/membrane/envelope biogenesis; [N] Cell motility; [O] Post-translational modification, protein turnover, and chaperones; [T] Signal transduction mechanisms; [U] Intracellular trafficking, secretion, and vesicular transport; [V] Defense mechanisms; [W] Extracellular structures; [Y] Nuclear structure; [Z] Cytoskeleton; [A] RNA processing and modification; [B] Chromatin structure and dynamics; [J] Translation, ribosomal structure and biogenesis; [K] Transcription; [L] Replication, recombination and repair; [C] Energy production and conversion; [E] Amino acid transport and metabolism; [F] Nucleotide transport and metabolism; [G] Carbohydrate transport and metabolism; [H] Coenzyme transport and metabolism; [I] Lipid transport and metabolism; [P] Inorganic ion transport and metabolism; [Q] Secondary metabolites biosynthesis, transport, and catabolism; [R] General function prediction only; [S] Function unknown; [X] Mobilome components. Groups are: *M. tuberculosis* (n=114), *M. africanum* (n=33), *M. bovis* (n=66), other animal strains (n=20). "Other animal strains" include: *M. caprae*, *M. mungi, M. pinnipedii*, *M. orygis*, *M. microti*, and "dassie bacillus".

We performed enrichment analyses of the COG categories of core, accessory, and strain-specific genomes each bacterial group against the core, accessory, and strain-specific genomes of the MTBC as a whole, respectively (Figure 2). In all bacterial groups, the category S, of proteins

with unknown function, was the top-enriched category in the core genome. Another commonly enriched category in all bacterial groups was transcription (K) (Figure 2).

In the accessory genome, except in *M. africanum*, the cellular motility category (N category) was the top enriched category in all bacterial groups. In *M. africanum*, the only enriched category of the accessory genome was unknown function (S). The category related to unknown function (S) stands out second in all other bacterial groups. In *M. tuberculosis*, *M. bovis* and “other animal strains”, this category is followed by the predominance of proteins related to lipid transport and/or metabolism (I) and replication, recombination, and repair (L). In *M. bovis*, proteins related to secondary metabolites biosynthesis, transport, and catabolism (Q) also stands out; this class is only enriched in the “animal strains”. The defense mechanism category (V category) is also well-represented in *M. tuberculosis* and *M. bovis*. Taken together, these results show that functional loss is not homogeneous among species of the MTBC.

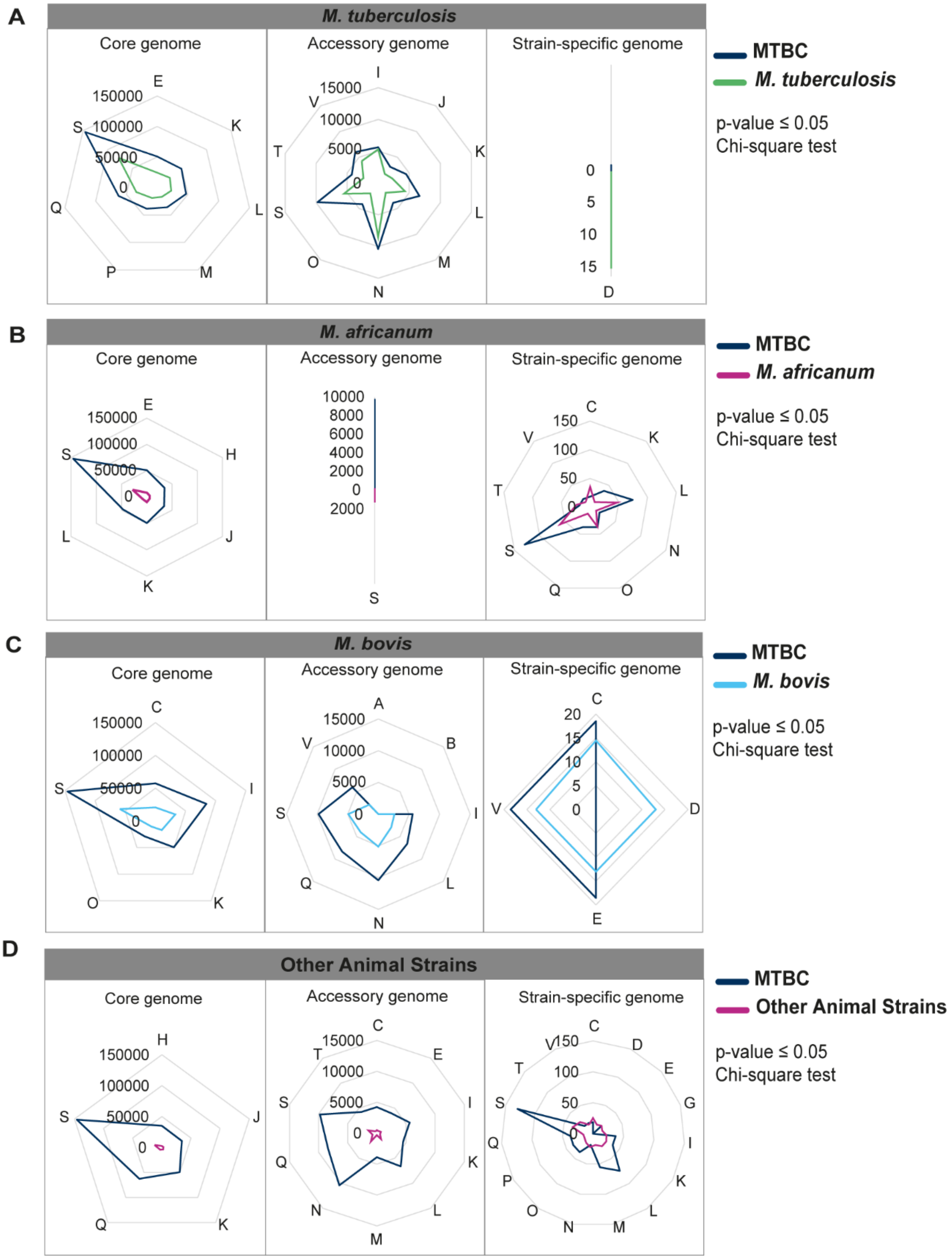

Figure 2. Enriched COG (Cluster of Orthologous Group) categories of the *Mycobacterium tuberculosis* complex (MTBC) groups compared to the MTBC as a whole with statistical significance with Pearson's chi-squared test. The spider chart represents the enriched proteins from each bacterial group versus proteins of the same functional categories present in the MTBC. COG Functional Categoryification. COG categories are: [D] Cell cycle control, cell division, chromosome partitioning; [M] Cell wall/membrane/envelope biogenesis; [N] Cell motility; [O] Post-

translational modification, protein turnover, and chaperones; [T] Signal transduction mechanisms; [U] Intracellular trafficking, secretion, and vesicular transport; [V] Defense mechanisms; [W] Extracellular structures; [Y] Nuclear structure; [Z] Cytoskeleton; [A] RNA processing and modification; [B] Chromatin structure and dynamics; [J] Translation, ribosomal structure and biogenesis; [K] Transcription; [L] Replication, recombination and repair; [C] Energy production and conversion; [E] Amino acid transport and metabolism; [F] Nucleotide transport and metabolism; [G] Carbohydrate transport and metabolism; [H] Coenzyme transport and metabolism; [I] Lipid transport and metabolism; [P] Inorganic ion transport and metabolism; [Q] Secondary metabolites biosynthesis, transport, and catabolism; [R] General function prediction only; [S] Function unknown; [X] Mobilome components. COG: Clusters of orthologous groups. MTBC: *Mycobacterium tuberculosis* complex. “Other animal strains” include: *M. caprae*, *M. mungi, M. pinnipedii*, *M. orygis*, *M. microti*, and “dassie bacillus”.

References

1. Soler-Camargo NC, Silva-Pereira TT, Zimpel CK, Camacho MF, Zelanis A, Aono AH, et al. The rate and role of pseudogenes of the Mycobacterium tuberculosis complex. Microb Genomics. 2022;8. doi:https://doi.org/10.1099/mgen.0.000876
2. Safi H, Gopal P, Lingaraju S, Ma S, Levine C, Dartois V, et al. Phase variation in Mycobacterium tuberculosis glpK produces transiently heritable drug tolerance. Proc Natl Acad Sci U S A. 2019;116: 19665–19674. doi:10.1073/pnas.1907631116
3. Pajuelo D, Tak U, Zhang L, Danilchanka O, Tischler AD, Niederweis M. Toxin secretion and trafficking by Mycobacterium tuberculosis. Nat Commun. 2021;12: 1–13. doi:10.1038/s41467-021-26925-1
4. Bespiatykh D, Bespyatykh J, Mokrousov I, Shitikov E. A Comprehensive Map of *Mycobacterium tuberculosis* Complex Regions of Difference. mSphere. 2021;6. doi:10.1128/MSPHERE.00535-21
5. Abuhammad A. Cholesterol metabolism: a potential therapeutic target in Mycobacteria. Br J Pharmacol. 2017;174: 2194. doi:10.1111/BPH.13694
6. Pawełczyk J, Brzostek A, Minias A, Płociński P, Rumijowska-Galewicz A, Strapagiel D, et al. Cholesterol-dependent transcriptome remodeling reveals new insight into the contribution of cholesterol to *Mycobacterium tuberculosis* pathogenesis. Sci Reports 2021 111. 2021;11: 1–16. doi:10.1038/s41598-021-91812-0
7. Marjanovic O, Iavarone AT, Riley LW. Sulfolipid accumulation in Mycobacterium tuberculosis disrupted in the mce2 operon. J Microbiol. 2011;49: 441–447. doi:10.1007/s12275-011-0435-4
